# Structures of *Neisseria gonorrhoeae* MtrR-operator complexes reveal molecular mechanisms of DNA recognition and antibiotic resistance-conferring clinical mutations

**DOI:** 10.1093/nar/gkab213

**Published:** 2021-03-30

**Authors:** Grace A Beggs, Julio C Ayala, Logan G Kavanaugh, Timothy D Read, Grace M Hooks, Maria A Schumacher, William M Shafer, Richard G Brennan

**Affiliations:** Department of Biochemistry, Duke University School of Medicine, Durham, NC 27710, USA; Department of Microbiology and Immunology, Emory University School of Medicine, Atlanta, GA 30322, USA; Department of Microbiology and Immunology, Emory University School of Medicine, Atlanta, GA 30322, USA; Department of Medicine, and the Emory Antibiotic Resistance Center, Emory University School of Medicine, Atlanta, GA 30322, USA; Emory Antibiotic Resistance Center, Emory University School of Medicine, Atlanta, GA 30322, USA; Department of Biochemistry, Duke University School of Medicine, Durham, NC 27710, USA; Department of Biochemistry, Duke University School of Medicine, Durham, NC 27710, USA; Department of Microbiology and Immunology, Emory University School of Medicine, Atlanta, GA 30322, USA; Emory Antibiotic Resistance Center, Emory University School of Medicine, Atlanta, GA 30322, USA; Laboratories of Bacterial Pathogenesis, VA Medical Center, Decatur, GA 30033, USA; Department of Biochemistry, Duke University School of Medicine, Durham, NC 27710, USA

## Abstract

Mutations within the *mtrR* gene are commonly found amongst multidrug resistant clinical isolates of *Neisseria gonorrhoeae*, which has been labelled a superbug by the Centers for Disease Control and Prevention. These mutations appear to contribute to antibiotic resistance by interfering with the ability of MtrR to bind to and repress expression of its target genes, which include the *mtrCDE* multidrug efflux transporter genes and the *rpoH* oxidative stress response sigma factor gene. However, the DNA-recognition mechanism of MtrR and the consensus sequence within these operators to which MtrR binds has remained unknown. In this work, we report the crystal structures of MtrR bound to the *mtrCDE* and *rpoH* operators, which reveal a conserved, but degenerate, DNA consensus binding site 5′-MCRTRCRN_4_YGYAYGK-3′. We complement our structural data with a comprehensive mutational analysis of key MtrR-DNA contacts to reveal their importance for MtrR-DNA binding both *in vitro* and *in vivo*. Furthermore, we model and generate common clinical mutations of MtrR to provide plausible biochemical explanations for the contribution of these mutations to multidrug resistance in *N. gonorrhoeae*. Collectively, our findings unveil key biological mechanisms underlying the global stress responses of *N. gonorrhoeae*.

## INTRODUCTION

The rise in multidrug resistance amongst clinical strains of *Neisseria gonorrhoeae*, the aetiological agent of gonorrhea, is considered to be an urgent public health threat by the Centers for Disease Control and Prevention (CDC) (1). Resistance to the last-line antibiotics azithromycin and ceftriaxone has been identified in clinical isolates across the world (2). Whole-genome sequencing of multidrug resistant gonococcal isolates revealed key mutations within the *mtrR* gene that contribute to and have been associated with multidrug resistance (3–7). The *mtrR* gene encodes a transcriptional repressor of the *mtrCDE* multidrug efflux transporter genes (8). Genetic evidence suggests antibiotic resistance-conferring mutations of MtrR, the product of the *mtrR* gene, often lead to the overexpression of the MtrCDE multidrug efflux system (6,9,10). Myriad cytotoxins are exported by this efflux system including hydrophobic drugs, dyes, bile salts, and the human antimicrobial peptide LL37 (11,12). However, the precise structural basis for the biochemical mechanisms by which these mutations confer resistance has not been described.

Recently, we demonstrated that MtrR specifically recognizes and binds to bile salts present at extra-genital infection sites of gonococci (13); this results in derepression of the *mtrCDE* efflux transporter genes. This is characteristic of classic inducer/repressor mechanisms or of one-component systems, which are common amongst TetR-family transcriptional regulators (TFRs) such as MtrR (14,15). TFRs are highly ubiquitous throughout bacteria and include many multidrug efflux regulators besides MtrR. Previously, we showed that MtrR adopts a typical TFR fold: MtrR is a functional homodimer composed of nine α-helices that form a C-terminal ligand-binding/dimerization domain and an N-terminal helix-turn-helix (HTH) DNA-binding motif (13). This HTH motif binds an operator sequence within the *mtrCDE* promoter (13,16).

Intriguingly, MtrR regulates several genes throughout the gonococcal genome in addition to the *mtrCDE* efflux transporter genes (17). For example, MtrR represses directly the expression of *rpoH*, an essential alternate sigma factor that is important for gonococcal oxidative stress responses; DNase protection assays revealed direct MtrR binding to the *rpoH* operator (17). In addition, MtrR appears to regulate *hsp33*, which is involved in peroxide and heat stress responses in *Neisseria gonorrhoeae*, *glnE*, which regulates glutamine biosynthesis, *glnA*, which encodes a glutamine synthetase, *gdhR*, a regulator of amino acid transport and L-glutamate dehydrogenase in *N. meningitidis*, *farR*, a negative regulator that controls the expression of the *farAB* fatty acid efflux pump, and the *ponA* and *pilQ*, genes, which encode penicillin-binding protein 1 and the outer membrane pilin secretin protein, respectively, and modulate gonococcal penicillin susceptibility (17–22). Such global gene control by MtrR contrasts with ‘local-only’ or ‘proximal’ transcription control observed for many structurally and biochemically well-characterized multidrug binding transcription regulators, such as MexR (23,24), CmeR (25), QacR (26), AcrR (27) and TtgR (28). Although genetic studies have identified MtrR target operators, the biochemical mechanisms by which MtrR recognizes different DNA-binding sites have not been determined. A major impediment to our understanding is the lack of an obvious MtrR-DNA binding consensus sequence.

Here, we report studies on MtrR to determine the structural and biochemical mechanisms of its DNA recognition and induction mechanisms. In this work, we describe the structures of the MtrR-*mtrCDE* and MtrR-*rpoH* operator complexes as well as *in vitro* and *in vivo* experiments to validate the structural mechanisms of MtrR-DNA binding and their physiological relevance. Furthermore, the comparison of these MtrR-DNA structures with induced MtrR provided fundamental insight into the conformational changes and structural dynamics of MtrR necessary for induction of the MtrR regulon. In addition, these structures allowed *in silico* modelling of frequently observed mutations in clinically relevant, multidrug resistant strains of *N. gonorrhoeae* thereby providing a plausible biochemical explanation for the contribution of these mutations to antibiotic resistance. Finally, understanding these mechanisms at a biochemical and structural level provides critical insight into the global stress response mechanisms of this pathogen that could usher in novel therapeutic strategies.

## MATERIALS AND METHODS

### Overexpression and purification of MtrR

MtrR was expressed and purified as previously described with the following modifications to the protein purification protocol (13,16). Both wild-type (WT) and point mutants of MtrR were expressed using the pMCSG7 vector and were in-frame with the N-terminal hexa-histidine tag and the tobacco etch virus (TEV) protease cleavage site. Cell pellets resuspended in 50 ml of buffer A (20 mM Tris–HCl [pH 8.0], 200 mM NaCl, 10% glycerol and the reductant 1 mM tris-2-carboxyethyl phosphine hydrochloride [TCEP]) were lysed by sonication (Fisher Scientific). Upon initial purification of the protein from clarified lysate by Ni-nitrilotriacetic acid (NTA) affinity chromatography to >95% homogeneity, the hexa-histidine tag was cleaved by TEV protease digestion. Cleaved MtrR was purified from TEV protease by Ni-NTA affinity chromatography. Cleaved MtrR was further purified by size exclusion chromatography (S200 column) and concentrated to ∼28 mg/ml using an Amicon UltraCel-10 membrane filter. Selenomethionine-derivatized MtrR (Semet-MtrR) was overexpressed as previously described (13, 29) and purified as native MtrR.

### Crystallization, data collection and structure determination

Purified MtrR (native or Semet-MtrR) concentrated to ∼28 mg/ml was mixed with 1.1 mM 21-mer oligoduplex (see Supplementary Figure S1 for sequences) immediately before hanging drops were set-up for crystallization of MtrR-*mtrCDE* and Semet-MtrR-*rpoH* operator complexes by the hanging drop-vapor diffusion method. The crystallization solution for these complexes contained 200 mM calcium acetate, 27% polyethylene glycol MW-8000, and 100 mM Tris, pH 7.5. The crystals assumed the space group *C* 1 2 1; unit cell parameters are listed in Table 1. Crystals of the MtrR-*mtrCDE* and Semet-MtrR-*rpoH* operator complexes were flash frozen using 15% glycerol as a cryoprotectant. Single wavelength anomalous diffraction (SAD) data on the Semet-MtrR-*rpoH* operator complex were collected remotely under cryogenic conditions at the Advanced Light Source (ALS) on beam line 5.0.2; non-anomalous single-wavelength diffraction data of MtrR-*mtrCDE* operator complex were collected under cryogenic conditions on beam line 8.3.1. X-ray intensity data were processed with iMOSFLM (30) and SCALA (31). The MtrR-*rpoH* operator complex structure was solved by molecular replacement (MR)-SAD to 2.8 Å resolution with AutoSol (32); the input model was a single dimer of MtrR with sidechains removed and missing residues 28–51 (α2-α3) (edited from PDB ID: 6OF0). The MtrR-*rpoH* operator complex structure was then solved to higher resolution (2.6 Å) by molecular replacement using the structure determined by MR-SAD as the input model and Phaser (33). Note that although our higher resolution dataset of the MtrR-*rpoH* operator complex included Semet-MtrR, the anomalous signal for this crystal was very weak. Thus, we could not successfully perform *de novo* phasing methods with this dataset. The MtrR-*mtrCDE* operator complex structure was solved by MR to 2.7 Å resolution using the protein dimer from the MtrR-*rpoH* operator complex structure as the input model and Phaser. Iterative rounds of model building using COOT (34) and refinement and validation using Phenix (35–37) resulted in the final models of the MtrR-*mtrCDE* and MtrR-*rpoH* operator complexes which were visualized with PyMOL (38). Selected data collection and refinement statistics are summarized in Table 1.

**Table 1.** Selected crystallographic data and refinement statistics

	Semet-MtrR-*rpoH* (MR-SAD)	Semet-MtrR-*rpoH* (MR)	MtrR-*mtrCDE* (MR)
**Data collection and phasing**			
Unit cell: *a*, *b*, *c*	151.4, 58.1, 68.6	151.0, 58.1, 68.2	151.0, 58.1, 68.2
Unit cell: α, β, γ	90.0, 90.1, 90.0	90.0, 90.1, 90.0	90.0, 114.3, 90.0
Wavelength	0.9794	0.9793	1.1158
Resolution (Å)	75.63–2.80	50.61–2.60	69.84–2.70
*R*_merge_^a^	0.087 (0.507)^b^	0.071 (0.340)	0.071 (0.440)
*R*_meas_^c^	0.123 (0.697)	0.085 (0.406)	0.088 (0.577)
Mean *I*/σ*I*	9.0 (2.0)	6.9 (3.3)	7.7 (1.8)
CC(1/2)	0.997 (0.795)	0.992 (0.920)	0.996 (0.833)
Completeness	96.9 (96.6)	99.3 (98.0)	95.1 (79.4)
Multiplicity	4.1 (3.9)	3.3 (3.4)	2.7 (2.0)
Anomalous completeness	90.1 (90.1)	87.8 (86.3)	
Anomalous multiplicity	2.2 (2.0)	1.6 (1.8)	
No. selenium sites	6 (out of 8 total)		
Overall figure of merit^d^	0.443		
**Refinement statistics**			
*R*_work_/*R*_free_^e^ (%)		19.9/25.6	22.8/28.2
No. protein atoms		3125	3129
No. DNA atoms		855	855
*B* factors macromolecule (Å^2^)		52.34	81.96
No. of calcium ions		8	4
Solvent no.		68	69
Ramachandran favored/allowed (%)		97.0/3.0	95.5/4.25
**RMSD**			
Bond lengths (Å)		0.003	0.002
Bond angles (°)		0.550	0.485

^a^ *R*_merge_ = ΣΣ|*I*_*hkl*_– *I*_*hkl*(*j*)_|/Σ*I_hkl_*, where *I*_*hkl*(*j*)_ is the observed intensity and *I_hkl_* is the final average intensity value.

^b^Values in parentheses are for highest resolution shell.

^c^ *R*_meas_ = Σ[(*N*/*N* – 1)^1/2^] Σ|*I_hkl_*– *I*_*hkl*(*j*)_|/ Σ*I_hkl_*, where *I*_*hkl*(*j*)_ is the observed intensity and *I_hkl_* is the final average intensity value.

^d^Figure of Merit = <|Σ*P*(α)e^iα^/Σ*P*(α)|>, where α is the phase and *P*(α) is the phase probability distribution.

^e^ *R*_work_ = Σ||*F*_obs_| – |*F*_calc_||/Σ|*F*_obs_| and *R*_free_ = Σ||*F*_obs_| – |*F*_calc_||/Σ|*F*_obs_|; where all reflections belong to a test set of 5% randomly selected reflections.

### Fluorescence polarization-based DNA binding assay

Fluorescence polarization-based DNA-binding data were collected with a Panvera Beacon 2000 fluorescence polarization system (Invitrogen) and analyzed with Prism (GraphPad Software) as previously described with the following modifications to the experimental conditions (13). Increasing concentrations of purified MtrR were titrated into binding buffer (20 mM Tris–HCl [pH 7.5], 100 mM NaCl, 2.5% glycerol, 1 mM TCEP) containing 1 μg of bovine serum albumin (BSA), 1 μg of poly deoxyinosinic:deoxycytidylic (dI–dC) acid, and 1 nM of 5′-fluorescein-labelled DNA. BSA and poly(dI-dC) were included in these experiments to control for nonspecific protein–DNA interactions. The reported dissociation constants in Table 2 are averages from at least three independent experimental measurements.

**Table 2.** The dissociation constants of MtrR for selected wild type and mutated DNA-binding sites

Target oligoduplex	*K*_d_ (nM)^a^
*rpoH* 27mer	8 ± 1
*mtrCDE* 21mer	50 ± 4
*mtrCDE* 27mer	43 ± 4
ApY 27mer	293 ± 24
GpY 27mer	Nonspecific binding
*emrAB* 22mer	Nonspecific binding

^a^Reported values are averages and associated standard error of the mean from at least three separate experiments.

### Determination of apparent melting temperatures

To compare the structural stability of MtrR WT and A39T mutant, circular dichroism (CD) data were collected as a function of temperature and used to determine apparent melting temperatures (*T*_m_). The CD data were collected with an Aviv Spectrometer, model 435. Samples contained 1 μM protein in buffer composed of 20 mM Tris–HCl pH 7.5, 100 mM NaCl, 2.5% glycerol and 1 mM TCEP. The CD signal was monitored at 220 nm with a bandwidth of 1 nm. Data were collected at 1°C steps from 25°C to 90°C, with 60 s temperature equilibration and averaging times. Data were analyzed using the van’t Hoff analysis. The derivative of the slope at the midpoint of the unfolding transition was used to assess the apparent *T*_m_.

### Site-directed mutagenesis

To introduce point mutations into the *mtrR*-pMCSG7 construct, we performed DpnI-mediated site-directed mutagenesis as previously described (13).

### Strains and media

*Neisseria gonorrhoeae* strains used in this study are described in Supplementary Table S1. Gonococcal cultures were grown in a 37°C incubator under 5% (v/v) CO_2_ on GC agar plates supplemented with Kellogg's supplements I and II (39).

### Gonococcal genetic transformations

Plasmids and oligonucleotide primers used are described in Supplementary Table S2. *Neisseria gonorrhoeae* strains with single *mtrR* missense mutations were constructed in the parental FA19 Str^R^ strain. Briefly, 400 ng of DNA vectors pGAB027RGB (bearing *mtrR* R44A), pGAB028RGB (*mtrR* G45A) and pGAB029RGB (*mtrR* Y48F) were used to transform strain FA19 Str^R^ by homologous recombination to generate strains JC55, LK01 and LK02 respectively, using the spot agar transformation method described before (40). Transformants were selected on GC agar plates containing Erythromycin 0.5 μg/ml. To confirm the mutations, the *mtrR* coding sequence and the complete upstream intergenic region were amplified by PCR and sequenced using primers KH9#3 and CEL1 (Supplementary Table S2) and gDNA from the selected transformants.

### Extraction of total RNA and qRT-PCR

Gonococcal cultures were grown in GC broth at 37 °C in an orbital shaker (225 RPM) to late exponential phase. Samples (1 ml) were centrifuged and resuspended in 200 μL RNA*later* solution (Ambion) and incubated 10 min on ice; recentrifuged and cell pellets stored at –80 °C. Total RNA extraction was performed using the RNeasy mini kit (Qiagen) following the company's protocol. Genomic DNA contamination was removed using the Turbo DNA-free Kit (Invitrogen). DNase I-digested total RNA samples were reverse transcribed using the Quanti-Tect Reverse Transcription Kit (QIAGEN). Quantitative Real Time PCR (qRT-PCR) was conducted using the IQ SYBR Green Supermix Kit and a CFX Connect Real Time System (Bio-Rad Laboratories). Gene expression values were presented as the relative expression in the mutant normalized to the WT value (Normalized expression ratios) and were calculated using the ΔΔCt method (41) with the formula 2 – ([Ct GI(mutant) – Ct IR(mutant)] – [average(Ct GI(WT) – Ct IR(WT))]), where Ct is the fractional threshold cycle, GI is the gene of interest and IR is the internal reference gene. The level of *recA* (NGO0741) and *rmpM* (NGO1577) mRNAs were used as internal reference genes. The following primer pairs were used to quantify relative mRNA levels: recAqFw/recAqRv for *recA*, rmpM_qRT_F/rmpM_qRT_R for *rmpM*, mtrC_qRT_F/mtrC_qRT_R for *mtrC* (NGO1365), mtrR_qRT_F/mtrR_qRT_R for *mtrR* (NGO1366) and rpoH_qRT_F/rpoH_qRT_R for *rpoH* (NGO0288).

### Analysis of genomic data for clinical isolates of *N. gonorrhoeae*

Raw data of *N. gonorrhoeae* genomes sequenced by Ezewudo *et al.* (42) were reprocessed using the Bactopia pipeline (43). Complete MtrR protein sequences were retrieved from 55 genomes and aligned using MUSCLE (44). The alignments were further processed using Bioconductor functions to examine patterns of individual mutations (45). Twenty-three of these genomes were found to have a single base pair deletion upstream within the *mtrR* promoter known to repress *mtrR* transcription and elevate antibiotic resistance phenotypes due to increased levels of the MtrCDE efflux pump, identified by BLASTN (46) of a 29 bp probe sequence (47).

## RESULTS

### Structures of MtrR-*rpoH* and MtrR-*mtrCDE* operator complexes

**Figure 1.**
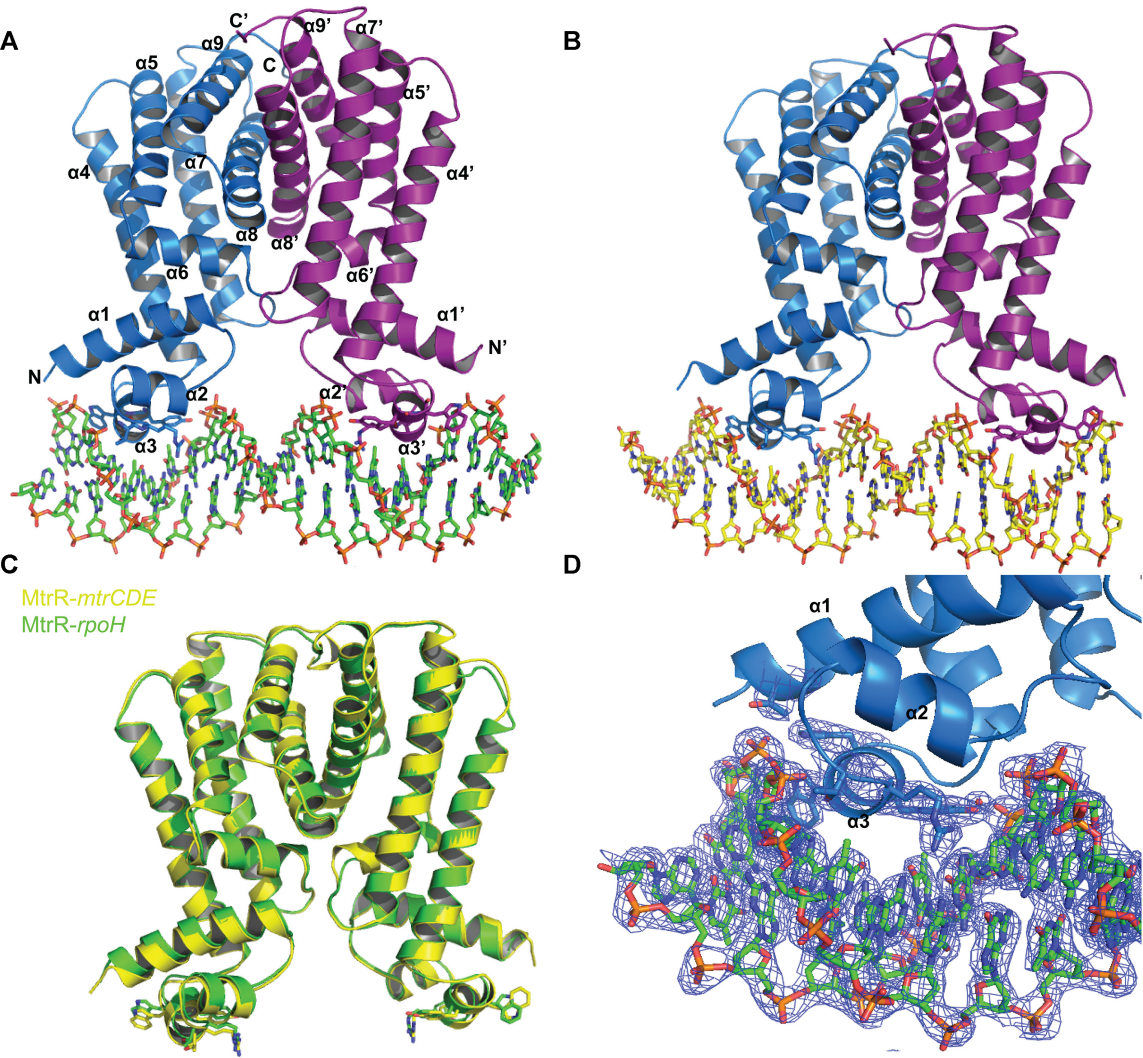
Overview of MtrR-DNA bound structures. (**A**) MtrR bound to the *rpoH* operator. The subunits of MtrR are shown in marine and purple; *rpoH* is shown in green. Secondary structure elements of MtrR are labelled. (**B**) MtrR bound to the *mtrCDE* operator, which is shown in yellow; MtrR is colored as in (**A**). (**C**) Overlay of MtrR from the MtrR-*rpoH* (green) and the MtrR-*mtrCDE* operator complex structures (yellow). (**D**) Closer view of DNA binding domain of MtrR in complex with the *rpoH* operator. The DNA and key contact residues are shown as sticks and the corresponding 2*F*_o_– *F*_c_ electron density map is contoured at 1.0 σ and shown in blue mesh.

MtrR was crystallized with a 21-basepair oligoduplex of the *rpoH* operator (17). The crystal structure of MtrR*-rpoH* complex was determined to 2.80 Å resolution by molecular replacement in combination with single-wavelength anomalous dispersion (MR-SAD) methods using Semet-MtrR. This structure was then used to solve the structure of a higher resolution Semet-MtrR-*rpoH* complex to 2.60 Å resolution by molecular replacement (MR) resulting in final *R*_work_ and *R*_free_ values of 19.9% and 25.6%, respectively, after refinement and model rebuilding (Table 1). In the structure, each subunit of the dimeric MtrR is composed of nine α-helices (Figure 1A). The residues comprising each helix are α1: 10–26, α2: 33–39, α3: 44–50, α4: 53–78, α5: 84–102, α6: 103–111, α7: 123–148, α8: 158–180, α9: 185–203. The DNA-binding domain consists of helices α1–α3, in which α3 is the DNA-recognition helix of the HTH motif (α2–α3). A four-helix bundle between α8–α9 and α8′–α9′ forms the vast majority of the dimerization interface between the two MtrR subunits that comprise the functional homodimer. The asymmetric unit of the MtrR-*rpoH* operator complex crystal structure contains a functional homodimer of MtrR bound to the double-stranded 21-mer encompassing the *rpoH* operator site (Figure 1A and Supplementary Figure S1). The root mean square deviation (RMSD) of the pairwise alignments of all corresponding Cα atoms of the two subunits in the asymmetric unit is 0.11 Å. Furthermore, the MtrR residues that contact the *rpoH* DNA are the same for each subunit. Additionally, the structural and helical parameters of the oligoduplex adhere to those for B-DNA with regard to twist, slide, tilt, roll, and shift (48). However, the average major groove width is 12.5 Å compared to the average major groove width of 11.4 Å for ideal B-DNA. Therefore, the major grooves of the DNA are slightly widened, which likely occurs upon formation of the complex when α3 and α3′ insert into adjacent major grooves of the DNA (Figure 1A).

The MtrR-*mtrCDE* operator complex was crystallized under the same conditions as the MtrR-*rpoH* operator complex and was solved to 2.70 Å resolution by MR with final *R*_work_ and *R*_free_ values of 22.8% and 28.2%, respectively (Table 1). As with the MtrR-*rpoH* operator complex, the asymmetric unit of the MtrR-*mtrCDE* operator complex crystal consists of a single MtrR dimer bound to the double-stranded 21-mer of the *mtrCDE* operator (Figure 1B and Supplementary Figure S1) (8). The RMSD corresponding to the alignment of the two MtrR subunits in the MtrR-*mtrCDE* operator complex is 0.44 Å. With regard to the DNA, the twist, slide, roll, and shift between successive base pairs and the helical parameters match those of B-DNA. However, some successive base pairs contacted by MtrR in the MtrR-*mtrCDE* operator complex exhibit a negative tilt (average of –1.75°) compared to B-DNA (average of –0.01°) and the MtrR-*rpoH* complex (average of 0.20°) (48). As observed in the MtrR-*rpoH* operator complex, the major groove of the DNA is slightly widened compared to B-DNA with an average width of 12.6 Å. In addition, the MtrR residues that contact the DNA in the MtrR-*mtrCDE* operator complex are the same for both subunits with α3 and α3′ inserted into adjacent major grooves of B-DNA (Figure 1B). Alignment of the MtrR dimers from each structure reveals that their conformations are essentially identical with an RMSD of 0.49 Å (Figure 1C).

### MtrR–DNA contacts

The MtrR residues that contact *rpoH* as well as *mtrCDE* are localized to the recognition helices, α3 and α3′, the turn of the HTH motif, and the amino terminus of helix α1 (Figure 1D and Supplementary Figure S2). The stabilization of α3 within the major groove is effected by the ‘positioning’ helix (49), α2, which has its positively charged N-terminus pointed directly at the DNA phosphate backbone, and the hydroxyl groups of the sidechains of residues T43 and Y48, which make hydrogen bonds with the phosphate backbone (Supplementary Figure S2 and S2A). In addition, a conserved water molecule in both structures mediates contacts between R44, Y48, and the phosphate backbone.

**Figure 2.**
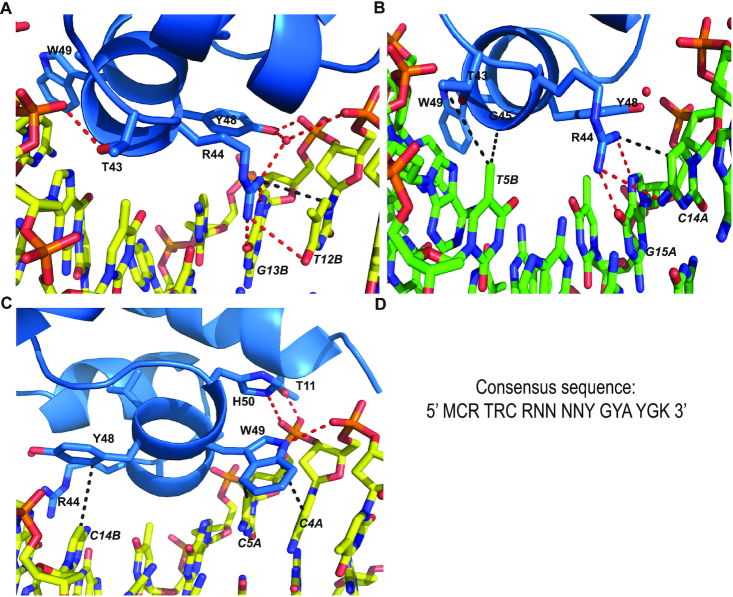
MtrR-DNA contacts and DNA-binding consensus sequence. (**A**) Interactions between MtrR and the 5′-TpG-3′ step and the phosphate backbone within the *mtrCDE* operator (yellow). Sidechains of key contact residues of MtrR (marine) are displayed as sticks. H-bonds are shown as dashed red lines and van der Waals contacts are shown in black dashed lines. (**B**) Contacts between MtrR (marine) and the 5′-CpG-3′ step as well as a thymine base within the *rpoH* operator (green). Sidechains and intermolecular interactions are denoted as in (A). (**C**) Direct contacts between T11, Y48, W49 and H50 and the *mtrCDE* operator (yellow). Sidechains and intermolecular interactions are indicated as in (A) and (B). (**D**) The structure-derived consensus sequence recognized by MtrR.

**Figure 3.**
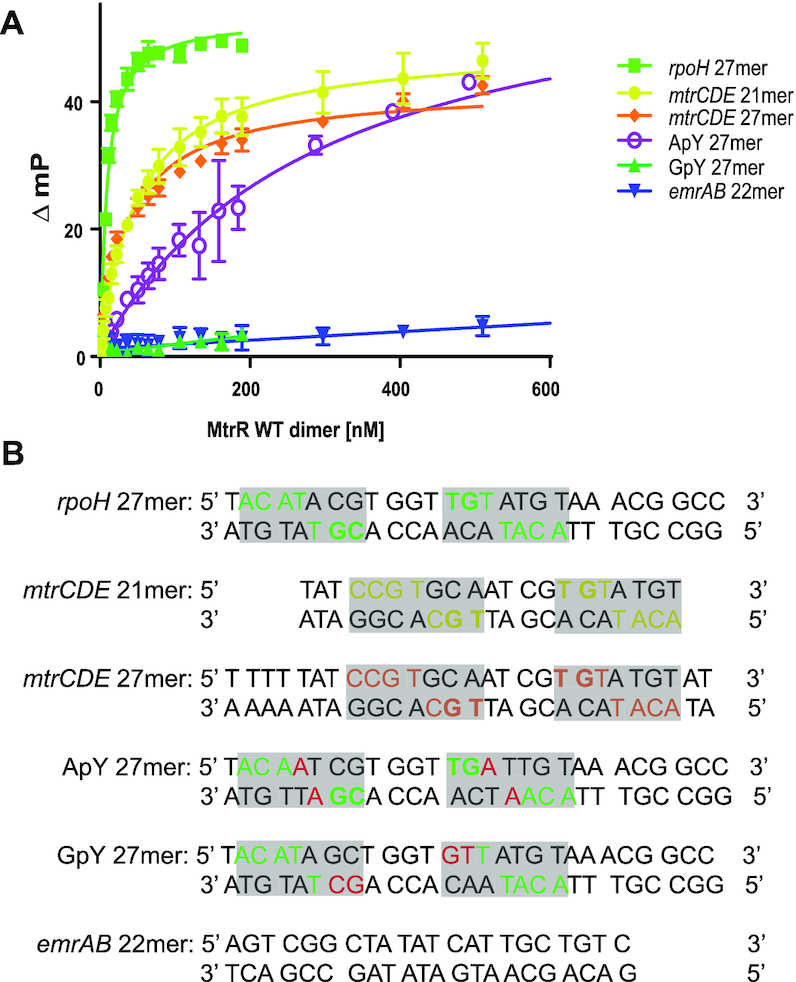
DNA-binding activity of MtrR *in vitro*. (**A**) Fluorescence polarization-based DNA binding assay data with MtrR WT and fluorescein-labelled oligoduplexes. The hyperbolic binding isotherms plot the change in millipolarization versus the MtrR dimer concentration are shown for each target oligoduplex: *rpoH* 27mer (lime green), *mtrCDE* 21mer (yellow), *mtrCDE* 27mer (orange), ApY 27mer (purple), GpY 27mer (kelly green), and *emrAB* 22mer (blue). (**B**) The sequences corresponding to the assayed oligoduplexes; the specific bases contacted by MtrR are highlighted in each oligoduplex with the corresponding colour in (A) with the exception of the ApY and GpY 27mer whereby the swapped 5′-ApY-3′ and 5′-GpY-3′ step is highlighted in red and the bases that could be contacted by MtrR are highlighted in green.

Five residues, T43, R44, G45, Y48 and W49, contact the DNA bases directly in both the MtrR-*rpoH* and the MtrR-*mtrCDE* operator structures. Critical to the DNA recognition of each operator site, the side chain of residue R44 contacts dyad-related 5′-YpG-3′ motifs, where Y is typically a thymine. By hydrogen bonding to N7 and O6 of the guanine base and making van der Waals contacts with the C7 or C5 atoms of the 5′ pyrimidine (50), R44 makes specific contacts with these bases. Additionally, 5′-YpG-3′ steps commonly exhibit unstacked bases (50); this is observed in our structures as evidenced by minor deviations from B-DNA parameters in the roll, slide, tilt or shift for these 5′-YpG-3′ motifs specifically. This unstacking of the bases allows the side chain of R44 to stack with the 5′ pyrimidine. Thus, R44 participates in both direct and indirect readout of these dinucleotide steps. In the MtrR-*mtrCDE* operator structure, both subunits of MtrR specifically recognize 5′-TpG-3′ steps at positions 15Ap16A and 12Bp13B. (Figure 3A and Supplementary Figure S2); however, in the MtrR-*rpoH* operator structure, one subunit recognizes a 5′-TpG-3′ step at position 13Bp14B and the other recognizes a 5′-CpG-3′ step at position 14Ap15A, again where stacking between the R44 side chain and cytidine is maintained (Figure 2B and Supplementary Figure S2). Other residues that participate in direct readout of the DNA sequence include T43 and G45, both of which make van der Waals interactions with the methyl group of a thymine base at positions 6A and 5B in the MtrR-*rpoH* operator structure (Figure 2B and Supplementary Figure S2) and 7A and 4B in the MtrR-*mtrCDE* operator structure (Supplementary Figure S2). Further, the aromatic side chains of Y48 and W49 engage in T-shaped π-π interactions with the DNA bases (Figure 2C). Specifically, Y48 contacts T16A and T15B within the *rpoH* operator and T17A and C14B within the *mtrCDE* operator; W49 contacts A3A, C4A, and A2B within the *rpoH* operator and with C4A, C5A, A1B and C2B within the *mtrCDE* operator (Supplementary Figure S2). In one subunit of the MtrR-*mtrCDE* operator complex structure, the side chain of residue W49 also engages in a hydrogen bond with the phosphate backbone (Figure 2C). Lastly, the side chains of two additional residues from each subunit form hydrogen bonds with the phosphate backbone in both the MtrR-*rpoH* and MtrR-*mtrCDE* operator complex structures; these are T11 from α1 and H50 from α3 (Figure 2C and Supplementary Figure S2). All contact distances shown in Figure 2 are listed in Supplementary Table S3.

The interactions between MtrR and the *mtrCDE* and *rpoH* operators reveal a previously unknown consensus recognition sequence (Figure 2D). Beyond the key interactions with the dyad-related 5′-YpG-3′ steps recognized directly by R44, the identity of the bases that are 3′ to the guanine of these steps is critical and is composed of a YpR dinucleotide step. Because 5′-YpR-3′ steps are more flexible than other dinucleotide steps (50), this 5′-YpR-3′ step may be necessary to allow DNA contact by Y48, which as noted engages in T-shaped π–π interactions with the pyrimidine at this position ($ \le$4.8 Å). Indeed, replacing this pyrimidine with a purine *in silico* results in the loss of the Y48-base interaction with the closest distance between the purine base and Y48 side chain >5.0 Å. Furthermore, this 5′-YpR-3′ step must specifically be a 5′-YpA-3′ step, which allows residues T43 and G45 to make specific contacts to the methyl group of the thymine base on the complementary strand. The base that is 5′ to this thymine on the complementary strand is always a purine. Changing this purine to a thymine generates a clash between that base and G45. Lastly, W49 makes direct contacts with the 5′-MpC-3′ step that is 5′ to this purine. Thus, within the target operator sites of both *rpoH* and *mtrCDE*, there are fourteen base-MtrR contacts that unveil the previously unrecognized MtrR-binding consensus sequence, 5′-MCRTRCRN_4_YGYAYGK-3′, where M signifies A or C; K, G or T; and N, any nucleotide (Figure 2D). The identities of the four base pairs connecting the inverted repeats of these cognate DNA sites are not conserved.

### Induction of MtrR regulated genes

The structures of two MtrR-DNA complexes and the ‘induced’ MtrR structure (13) allow the elucidation of the conformational changes that occur upon DNA-binding and MtrR induction. Because MtrR takes the same conformation in both the MtrR*-rpoH* and MtrR-*mtrCDE* operator structures, we limit our comparison to an induced form of MtrR (PDB ID: 6OF0) (13) and the higher resolution MtrR-*rpoH* complex structure (Supplementary Figure S3, Supplemental Video S1). The corresponding Cα atoms of the individual protomers of the induced and DNA-bound form of MtrR can be superposed with an RMSD of 2.1 Å, which indicates there is a significant difference between these two conformations. The most significant changes are associated with the motion of the DNA-binding domain (α1–α3). When helices α8-α9 are aligned and fixed for the two conformations, we observe a 20° rotation of the DNA-binding domain, which is shifted downward towards the center of the MtrR dimer in the DNA-bound conformation (51); the N-terminus of α1 alone shifts downward so that α1 is at a 21° slope in the DNA-bound form, likely stabilized by the T11-phosphate backbone interaction, when compared to the induced form (Supplementary Figure S3). The movement of the DNA-binding domain results in a closer center-to-center distance between the recognition helices of the HTH motifs in the dimer-bound DNA complex (37 Å) in sharp contrast to the same distance of the induced dimeric form (45 Å) and clearly is compatible with the ability of the recognition helices, α3 and α3′, to fit into successive major grooves of the B-DNA. In addition to this shift in the DNA-binding domain, α4 shifts with a 3.0 Å translation in the direction of the N-terminus of α4 and a 22° rotation of the central axis of the helix as it transitions from the induced to the DNA-bound form (Supplementary Figure S3), consequently bringing α4 slightly closer to α5 in the DNA-bound form. Another conformational change that occurs is the movement of α7, which must rotate 6° so that its N-terminus is pointing closer to α4 in order to accommodate the shift of the HTH motifs toward the center of the MtrR dimer dyad axis relative to their positions in the induced form.

### MtrR binds *rpoH* with higher affinity than *mtrCDE*

The structures of the MtrR-*rpoH* and MtrR-*mtrCDE* operator complexes reveal the specific and conserved sequences that MtrR recognizes within these operators. To determine the affinity and specificity of MtrR for these sites, we performed fluorescence polarization-based DNA binding assays (Figure 3A). We determined the binding affinity of MtrR to several DNA sequences including the oligoduplex containing the *rpoH* recognition site identified in the crystal structure (*rpoH* 27mer) as well as the *mtrCDE* binding site from the crystal structure (*mtrCDE* 21mer) (Figure 3B). Intriguingly, we observed nearly a ∼6-fold higher affinity of MtrR for *rpoH* than *mtrCDE* (Table 2). The measured dissociation constants corresponding to MtrR binding the *rpoH* and *mtrCDE* operators were ∼8 and ∼50 nM, respectively. Because of the difference in length of these two oligoduplexes, we also included a 27mer of the *mtrCDE* target site in our assays containing the genomic sequence previously identified in the literature (8) (*mtrCDE* 27mer). The dissociation constant we measured for MtrR binding to the *mtrCDE* 27mer was consistent with the *mtrCDE* 21mer dissociation constant (Table 2). Thus, the observed difference in affinity between *rpoH* and *mtrCDE* is not due simply to oligoduplex length. The difference in affinity is likely due to the difference in the identity of the pyrimidine in the 5′-YpG-3′ and 5′-YpA-3′ steps that MtrR recognizes. Indeed, comparing the contacts of MtrR to the pyrimidine in the 5′-YpA-3′ steps within the *rpoH* and *mtrCDE* operators reveals shorter distances between these interactions in the MtrR-*rpoH* operator complex. The sequence of the (N_4_) DNA that links the two halves of the binding sites is highly unlikely to contribute to the binding affinity differences as it is not contacted by MtrR and shows identical conformations in both structures ruling out indirect readout.

To test the specificity of MtrR for the *mtrCDE* and *rpoH* target sites, we assayed a ‘negative control’ operator sequence, that of the *emrAB* operon, which encodes the EmrAB efflux transporter system in *E. coli* and is repressed directly by the MarR family member, MprA (52,53). We observed no specific binding of MtrR to the *emrAB* operator oligoduplex and were unable to determine a dissociation constant (Figure 3 and Table 2). These data confirm the specificity of MtrR for the *mtrCDE* and *rpoH* operators. To underscore the importance of the 5′-YpG-3′ and subsequent 5′-YpA-3′ motifs for DNA recognition and high affinity binding by MtrR, we tested the binding of this repressor to an oligoduplex in which the 5′-YpG-3′ steps of the *rpoH* operator site were replaced by 5′-GpY-3′ steps (GpY 27mer, Figure 3B) as well as to an oligoduplex in which the 5′-YpA-3′ steps of the *rpoH* site were replaced by 5′-ApY-3′ steps (ApY 27mer, Figure 3B). As found for the *emrAB* DNA site, no specific binding was observed between MtrR and the GpY 27mer, thus underscoring the critical importance of the 5′-YpG-3′ steps in specific DNA binding by MtrR (Figure 3A and Table 2). In addition, there was >36-fold reduction in affinity between MtrR and the ApY 27mer compared to the native *rpoH* sequence; this substantiates the necessity of the 5′-YpA-3′ steps for high affinity DNA binding by MtrR (Figure 3A and Table 2).

### Validating DNA recognition mechanisms of MtrR

To validate the DNA recognition mechanisms observed in our crystal structures and to test the physiological relevance of these biochemical mechanisms, we generated a series of point mutants of MtrR residues that make contacts with the *rpoH* and *mtrCDE* operators. These point mutants included T11A, T43A, T43S, R44A, G45A, Y48F, W49F, W49A and H50A. As expected, these mutants eluted at the molecular weight of a dimer, as observed for WT MtrR, indicating that these point mutations did not affect the global fold or oligomerization state of the protein (Supplementary Figure S4). Thus, we infer that any changes in DNA-binding associated with these mutants are a direct result of the change in the specific residues assayed as opposed to global structural changes.

Utilizing our fluorescence polarization-based DNA binding assay, we performed a comprehensive study of the DNA-binding capabilities of our DNA-binding point mutants to both the *rpoH* and *mtrCDE* operator sequences (Supplementary Figure S5 and Table 3). Somewhat surprisingly, the binding affinities between the T11A mutant and DNAs (Supplementary Figure S5A) were reduced by ∼20–50-fold (Table 3), revealing the critical nature of the phosphate backbone contacts made by this residue. To the best of our knowledge, this is the only example of a direct DNA contact from the side chain of a residue located on α1 of a TFR suggesting an important role in the stabilization of the HTH motif on cognate DNA. Mutation of T43 to alanine completely abolished binding of MtrR to both the *rpoH* and *mtrCDE* operators (Supplementary Figure S5B) revealing the necessity of this residue for binding. However, mutation of T43 to serine only decreased the affinity and specificity of MtrR (Supplementary Figure S5C). Whilst the binding between MtrR(T43S) and the target operators (*rpoH* and *mtrCDE*) was weakened compared to WT binding, increased nonspecific binding was observed between this mutated protein and the control operators (*emrAB* and GpY). Thus, the methyl group of T43 appears to be critical for specific high affinity DNA-binding. Similar to the MtrR(T43A) mutant, MtrR(R44A) did not bind the target or control operators (Supplementary Figure S5D); these data confirm the absolute necessity of the R44 and the 5′-YpG-3′ motifs interactions for DNA-binding. MtrR(G45A) was not capable of binding *rpoH* specifically (Supplementary Figure S5E). Although a *K*_d_ for MtrR(G45A)-*mtrCDE* could be measured, these data correspond to only a minimal change in polarization that is comparable to the change in polarization when no binding event is observed (such as in the experiments with the T43A and R44A mutants). Thus, it is unlikely these data represent specific binding. Introducing the Y48F mutation abolished specificity in MtrR-DNA binding; in the absence of the hydrogen bonds made between Y48 and the phosphate backbone, highly robust nonspecific binding was observed (Supplementary Figure S5F). The dissociation constants associated with MtrR(W49F) binding to *rpoH* and *mtrCDE* were 6–8-fold higher than those associated with WT binding (Table 3). In addition, increased nonspecific binding between MtrR(W49F) and the control operators (*emrAB* and GpY) was observed (Supplementary Figure S5G). Eliminating the possibility of T-shaped π–π interactions between residue 49 and the DNA by mutating W49 to alanine terminated specific binding whereas nonspecific binding between MtrR(W49A) and all operators was observed (Supplementary Figure S5H). Lastly, elimination of the contact between the phosphate backbone and H50 resulted in a decreased DNA binding affinity of 20–47-fold (Table 3 and Supplementary Figure S5I); intriguingly, these data demonstrate that despite contacting the same phosphate group of the DNA, T11 and H50 are not able to compensate for each other in recognizing the phosphate backbone. Collectively, the binding data confirm the necessity of the MtrR-DNA contacts described by our structural analyses for specific, high affinity binding by MtrR to both the *mtrCDE* and *rpoH* operators.

**Table 3.** The dissociation constants of MtrR DNA-binding domain point mutants and selected DNA binding sites

	*K*_d_ (nM)^a^								
MtrR mutants	T11A	T43A	T43S	R44A	G45A	Y48F	W49F	W49A	H50A
Oligoduplexes									
*rpoH* 27mer	441 ± 37	NDB^b^	860 ± 180	NDB	NS^c^	NS	53 ± 3	NS	376 ± 35
*mtrCDE* 21mer	1076 ± 93	NDB	NS	NDB	(52 ± 13)^d^	NS	380 ± 44	NS	990 ± 100
GpY 27mer	NS	NDB	NS	NDB	NDB	NS	NS	NS	NS
*emrAB* 22mer	NS	NDB	NS	NDB	NDB	NS	NS	NS	NS

^a^Reported values are averages and associated standard error of the mean from three separate experiments.

^b^NDB = No detectable binding indicates that the data could not be fit by our binding equations.

^c^NS = Nonspecific binding is designated for interactions with *K*_d_ > 1.5 μM.

^d^The measured *K*_d_ is unreliable given small change in mP; it is unlikely these data represent specific binding.

### *In vivo* significance of *mtrR* point mutations

We next assessed the *in vivo* significance of certain point mutations for their ability to elevate macrolide and cationic antimicrobial peptide resistance and impact select gene expression in *N. gonorrhoeae*. For this purpose, plasmids bearing the A39T, R44A, G45A and Y48F *mtrR* point mutations were used in transformation experiments to replace the WT *mtrR* gene in antibiotic-sensitive strain FA19. Plasmids with the R44A, G45A and Y48F mutations readily transformed strain FA19 for increased (2-fold) resistance to erythromycin [Ery], while the A39T plasmid failed to do so. DNA sequencing of recovered transformants confirmed the presence of the selected point mutation and verified that it was the only change within the *mtrR* coding sequence or promoter region (data not presented). Regardless of the point mutation, all transformants exhibited two-fold increases in resistance to macrolides (azithromycin [Azi] and Ery) and a cationic antimicrobial peptide (polymyxin B [PMB]) as assessed using the agar dilution method; see Supplementary Table S4. In this respect, the point mutants resembled a previously reported transformant strain (JF1) containing a deletion of *mtrR* (17). A population analysis of antimicrobial susceptibility recently used to assess differences in antimicrobial susceptibility displayed by isogenic gonococci with distinct mutations impacting the structure of the MtrD transporter protein (54) confirmed that the MtrR point mutants were more resistant than the parental strain bearing the WT gene to the test antibiotics (Supplementary Figure S6).

**Figure 4.**
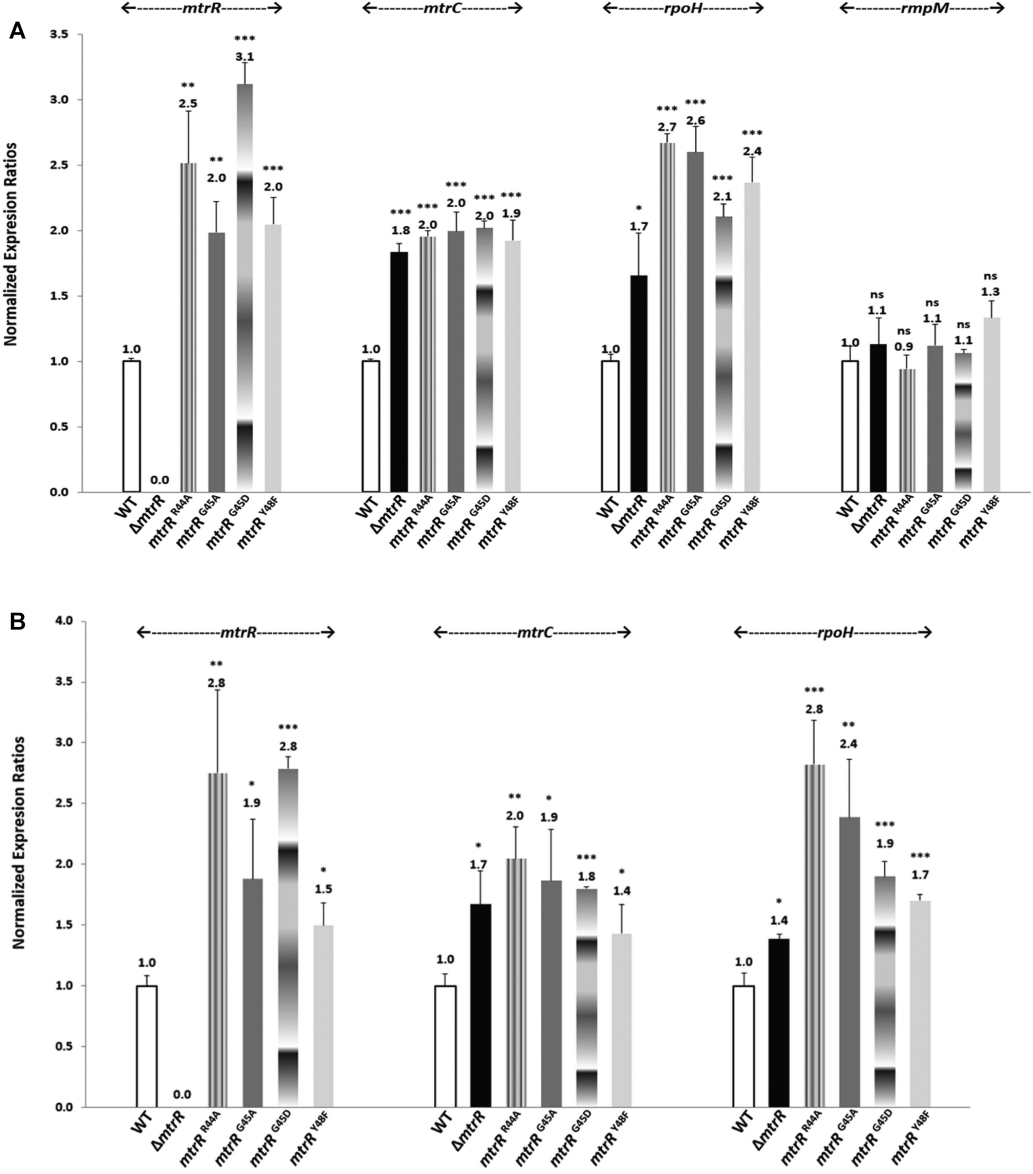
Effect of *mtrR* missense mutations on MtrR-regulated gene expression. *N. gonorrhoeae* WT FA19 Str^R^ and mutant strains JF1 (Δ*mtrR*), JC55 (*mtrR* R44A), LK01 (*mtrR* G45A), KH16 (*mtrR* G45D) and LK02 (*mtrR* Y48F) were grown in GC broth to late-logarithmic phase before RNA extraction. Data are represented as the mean + SEM of at least 4 biological and 3 technical samples. (**A**) Gene expression was normalized to WT levels as described in Materials and Methods using *recA* mRNA as internal reference. Statistics: Two-tailed t-Test of WT vs mutant (* *P* < 0.05, ** *P* < 0.01, **** *P* < 0.001, ns: non-significant). (**B**) Gene expression was normalized to WT levels as described in Materials and Methods using *rmpM* mRNA as internal reference. Statistics: One-tailed *t*-test of WT versus mutant (*P < 0.05, ** *P* < 0.01, **** *P* < 0.001, ns: non-significant).

We next assessed whether the different point mutations might differentially impact expression of MtrR-regulated genes. Quantitative reverse transcriptase-polymerase chain reaction (qRT-PCR) was used to measure transcripts for the reported MtrR-regulated genes *mtrR*, *mtrC* and *rpoH*. Point mutants R44A, G45A and Y48F expressed significantly similar elevated levels of *mtrC* and *rpoH* expression when compared to the WT strain (Figure 4).

### Biochemical effects of clinical mutations

**Figure 5.**
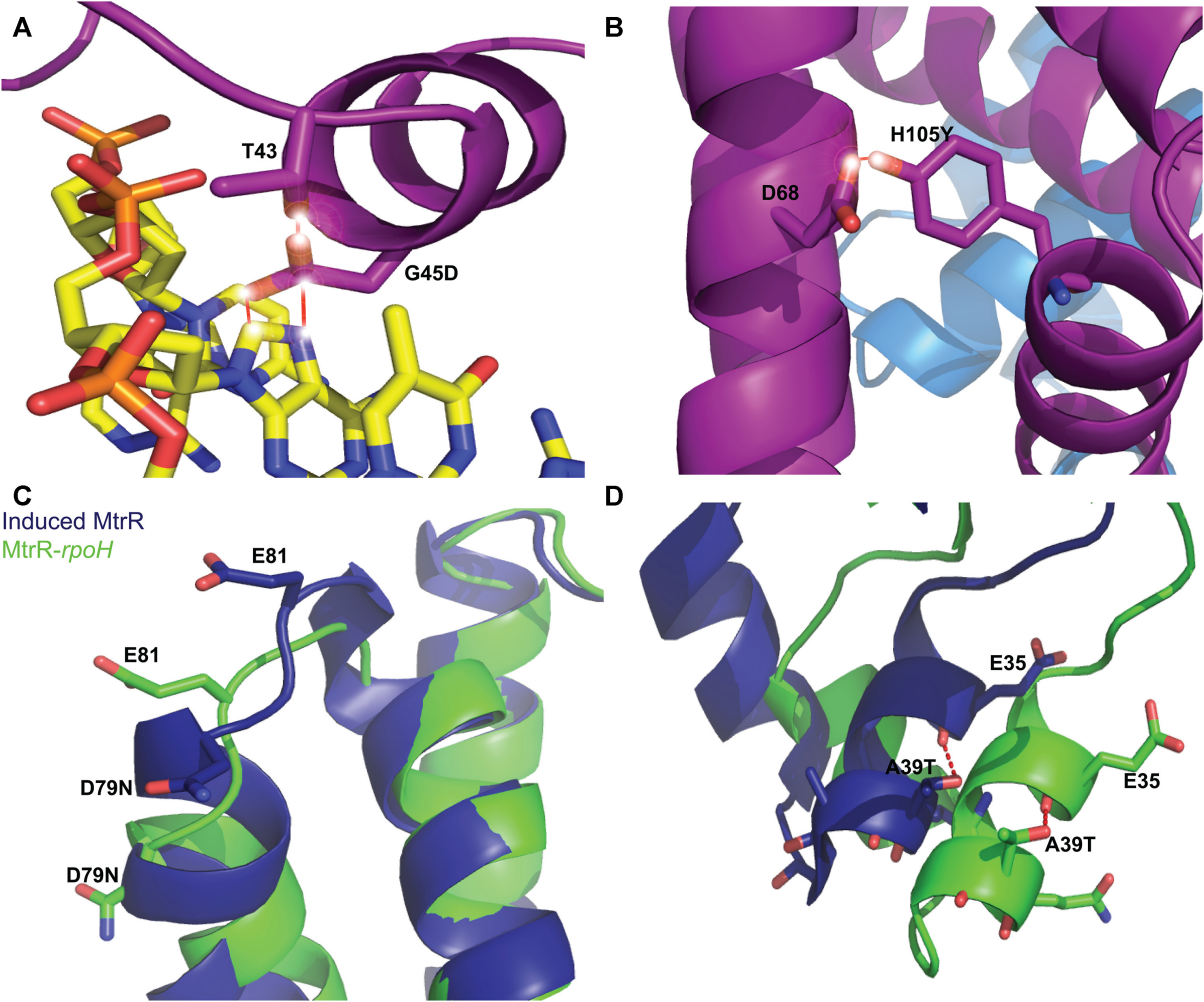
Biochemical mechanisms of clinical mutations conferring drug resistance. Modelling of the mutations G45D (**A**) and H105Y (**B**) in the MtrR-*mtrCDE* structure; steric clashes between the mutated sidechain and neighbouring bases or residues are indicated by the solid red lines and flare points. Modelling of the D79N (**C**) and the A39T (**D**) mutations in the induced (deep blue) and DNA-bound (green) conformations of MtrR. Hydrogen bonds are shown by red dashed lines.

The elucidation of the DNA recognition mechanisms of MtrR for its *rpoH* and *mtrCDE* target operators both *in vitro* and *in vivo* has allowed us to investigate further the biochemical effects of common mutations in drug-resistant clinical isolates of *N. gonorrhoeae*. In this respect, G45D, located at the N-terminus of the recognition helix, α3, was found in 20/55 clinical strains (42) (Supplementary Table S5) and has been associated with penicillin and macrolide resistance (4,5,55). *In silico* modelling of this mutation indicates that an MtrR(G45D) protein would be incapable of binding to its target DNA due to severe steric clash between the aspartate side chain and DNA as well as clash with the side chain of residue T43 in the DNA-bound conformation (Figure 5A). Furthermore, this mutation introduces a negative charge proximal to the anionic DNA phosphate backbone. A second commonly observed clinical mutation in the *mtrR* gene that is associated with drug resistance results in the MtrR(H105Y) protein (10,55,56) and is present in 26/55 isolates (42) (Supplementary Table S5). This residue is located in the ligand-binding/dimerization domain, on helix α6. Substitution with the larger tyrosine side chain introduces steric clash with the sidechain of D68 but only when MtrR is in the DNA-bound conformation (Figure 5B). It should be noted that severe clashes occur with all rotamers of G45D and H105Y in the DNA-bound conformation of MtrR. We also tested the DNA-binding activity of MtrR(G45D) and MtrR(H105Y) via our fluorescence polarization-based assay and found that MtrR(G45D) was incapable of binding DNA whilst the affinity of MtrR(H105Y) for DNA was reduced >12-fold compared to WT (Table 4, Supplementary Figure S7A and S7B). Accordingly, we assessed whether a G45D point mutant behaved similarly to the G45A point mutant described previously. As is shown in Supplementary Table S4, these mutants had a similar level of resistance to Azi, Ery and PMB. Further, both mutations similarly elevated expression of *mtrC* and *rpoH* (Figure 4). These mutations could contribute to resistance in gonococcal isolates in tandem with other *mtrR* point mutations as suggested by the occurrence of accompanying mutations in the HTH motif or *mtrCDE* operator in a recent survey of clinical isolates in the USA (57) (Supplementary Table S6).

**Table 4.** The dissociation constants of clinically relevant point mutants of MtrR binding to MtrR-selected DNA sequences

	*K*_d_ (nM)^a^			
MtrR mutants	A39T	G45D	D79N	H105Y
Oligoduplexes				
*rpoH* 27mer	31 ± 3	NDB^b^	NS^c^	340 ± 57
*mtrCDE* 21mer	170 ± 11	NDB	NDB	640 ± 130
GpY 27mer	NDB	NDB	NS	860 ± 80
*emrAB* 22mer	NDB	NDB	NS	NS

^a^Reported values are averages and associated standard error of the mean from three separate experiments.

^b^NDB = no detectable binding.

^c^NS = Nonspecific binding (*K*_d_ > 1.5 μM).

A common clinical mutation that the structures of MtrR provide somewhat more limited insight into its contribution to drug resistance is D79N (6). This mutation is found in 4/55 isolates (Supplementary Table S5) and is located on the loop between α4 and α5 (Figure 5C); much of the density surrounding this loop is not visible in either the induced or DNA-bound structures. However, helix α4 is critical to the induction mechanism of MtrR, whereby in the DNA-bound conformation the helix moves ‘down’ towards the DNA consequently allowing MtrR to adopt a high-affinity DNA-binding conformation (Supplementary Figure S3 and Supplemental Video S1). Thus, we would surmise that this allosteric mutation interferes with this movement and favors a drug-bound conformation in which α4 cannot move appropriately to effect high affinity DNA binding by helices α1, α2 and α3. Our DNA-binding assays support this supposition as MtrR(D79N) binds cognate DNA with significantly lower affinity, >10-fold higher *K*_d_, than WT protein (Table 4 and Supplementary Figure S7C).

Another clinical mutation within the MtrR HTH motif that we investigated is A39T (4,5,56). This mutation, present in 6/55 clinical isolates (42) (Supplementary Table S5), has been associated with resistance to azithromycin, penicillin, and tetracycline (7) and would allow hydrogen bonding with the main chain of α2 (Figure 5D). Because residue 39 is the C-cap of α2 in the DNA-bound conformation, this alanine-to-threonine mutation may alter the flexibility of the HTH motif as well as the stability of the protein. To interrogate this possibility further, we generated this point mutation and tested its relative stability by assessing its apparent melting temperature (*T*_m_) relative to WT and found a difference in *T*_m_ of ∼4°C (Supplementary Figure S8 and Supplementary Table S7). These data indicate the A39T mutant form of MtrR is less stable or that its α2 is not properly folded for optimal DNA binding compared to the WT. We also assessed the DNA-binding capabilities of the A39T mutant *in vitro* (Supplementary Figure S7D). This single point mutation caused a relatively mild reduction in DNA-binding affinity of ∼3- to 5-fold compared to WT (Table 4). Although only a modest loss of affinity, we tested whether this mutation could contribute directly to antibiotic resistance through the derepression of the *mtrCDE* efflux transporter genes. In contrast to other point mutations within the HTH motif, a plasmid containing A39T was unable to transform strain FA19 for increased macrolide resistance (data not presented), a finding consistent with its modest influence in MtrR affinity for the *mtrCDE* operator sequence. Accordingly, we hypothesize that any influence A39T might have on *mtrCDE* depression requires the presence of other MtrR amino acid changes common in clinical strains such as R44Q or Y48D substitution (4); as shown in our fluorescence polarization-based DNA binding studies, R44 and Y48 are critical for high affinity DNA binding (Table 3 and Supplementary Figure S5). This hypothesis is further supported by the high frequency of accompanying mutations in clinical isolates of *N. gonorrhoeae* containing the A39T mutation (57) (Supplementary Table S6).

## DISCUSSION

**Figure 6.**
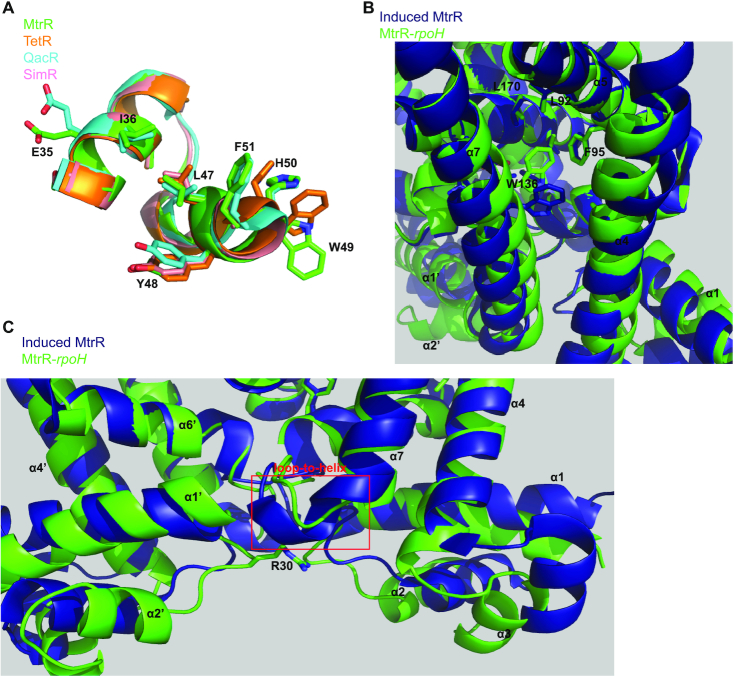
Structural insights into the MtrR Helix-Turn-Helix motif and MtrR induction mechanism. (**A**) Overlay of the Helix-Turn-Helix motifs of MtrR (green), TetR (orange), QacR (cyan), and SimR (pink). The sidechains of residues homologous to MtrR are displayed as sticks and labelled. (**B**) Overlay of the induced (deep blue) and DNA-bound (green) conformations of MtrR looking into the multidrug binding pocket. Selected residues protruding into the multidrug-binding pocket are labeled. (**C**) Overlay of the induced (deep blue) and DNA-bound (green) conformations of MtrR highlighting the loop-to-helix transition of residues 119–123 upon induction (highlighted by a red box). Key residue R30 is shown as sticks and labelled.

Here, we describe the DNA recognition mechanism of MtrR, a TFR and global gene regulator in *N. gonorrhoeae* (17). Interestingly, most TFRs are not global regulators (14,15), making MtrR an unusual member of this protein family. Nonetheless, the DNA-binding HTH motifs of MtrR and other TFRs are highly conserved as are their global folds. Despite this, the DNA-recognition and the induction mechanisms of only a few drug or multidrug/ligand-binding TFRs have been structurally characterized; amongst these are *Escherichia coli* TetR*, Staphylococcus aureus* QacR*, Streptomyces antibioticus* SimR, *Pseudomonas putida* TtgR and *Stenotrophomonas maltophilia* SmeT (26,28,58–62). Comparison of the HTH motifs between MtrR and the most thoroughly studied of these TFRs including TetR, QacR and SimR (Figure 6A) reveals the highest sequence homology between the MtrR HTH and the TetR and QacR HTH motifs with a sequence identity of 35%. However, the greatest structural homology is found between the MtrR HTH motif and the SimR HTH motif (RMSD = 0.42). The greatest similarity in the protein–DNA contacts across these different TFRs is a homologous tyrosine residue, Y48 in MtrR, that participates in π–π interactions with DNA base pairs. In all four proteins this conserved tyrosine also makes contacts with the phosphate backbone to buttress the base pair-specific contacts of other residues (58–60).

The availability of the DNA-bound and induced structures of these TFRs allows a fuller structural description of the induction mechanisms of all four of these TFRs. Out of the multidrug efflux regulators described, the induction mechanism of MtrR appears to most closely resemble that of QacR. Upon drug binding, residues 89–93 of QacR undergo a coil-to-helix transition resulting in relocation of α6; this, in turn, causes a rotation of the DNA-binding domain and a pendulum motion of α4 (26). Analogously, induction of MtrR is tied to a loop-to-helix transition of residues 119–123, which is linked to movement of α7, rotation of the DNA-binding domains, and rotation and translation of α4. In our previous work, we identified residue W136, which is located on α7, as a critical residue for induction of MtrR by chenodeoxycholate, a bile salt present at extra-genital gonococcal infection sites (13). In the DNA-bound structure, W136 appears to partly occlude the ligand-binding pocket (Figure 6B). Ligand-binding to MtrR results in the bending and rotation of α7 that relocates W136 to allow the ligand binding pocket to expand, and the loop-to-helix transition of residues 119–123 to occur (Figure 6B and C). As a result of this loop-to-helix transition, the DNA-binding domain of the other subunit (α1′-α3′) must rotate to avoid steric clash between residue R30, which is located on the loop between α1 and α2, and the N-terminus of α7. In addition to the HTH motif, α1 must also rotate to avoid steric clash between its C-terminus and the N-terminus of α7. This rigid-body motion of the DNA-binding domain is accompanied by the translation and rotation of α4, which is allowed by the pendulum motion of α7, as well as a slight rotation of α6 about itself so that the N-terminus is pointed more towards α4 whilst its C-terminus is pointed towards α7. As a result, the volume of the multidrug-binding pocket expands from ∼935 to ∼1520 Å^3^ (63). Ultimately, these structural changes result in an increased center-to-center distance of the recognition helices (α3-α3′) of ∼9 Å that is incompatible with binding to B-DNA, the conformation observed in the crystal structures of the MtrR-*rpoH* and MtrR-*mtrCDE* operator complexes.

It is worth noting that the structural changes of MtrR associated with the transition between the induced and DNA-bound forms also show some similarity to the induction conformational changes described for the TFR FadR from *Bacillus halodurans* (FadR_Bh_) (64) and FadR from *Sulfolobus acidocaldarius* (FadR_Sa_) (65), which both bind DNA to regulate fatty acid metabolism in their respective organisms. Upon DNA-binding, the DNA-binding domains (α1–α3) of FadR_Bh_ rotate decreasing the center-to-center distance of the HTH motifs; this rotation is accompanied by a pendulum-like motion of α4 in which the N-terminus of the helix is shifted towards the DNA-binding domain (64). Additionally, a kinking of α7 is observed so that its N-terminus rotates ∼10°. These conformational changes mimic those of MtrR, in which the primary movements include a rigid-body rotation of the DNA-binding domain, the re-orientation of α4 so that its N-terminus is pointed toward the DNA-binding domain in its DNA-bound form, and a rotation shifting the N-terminus of α7.

In addition to determining the structural mechanism of induction of MtrR, we have identified a consensus sequence that MtrR recognizes within the *mtrCDE* and *rpoH* operators. Intriguingly, this sequence, 5′ MCRTRCRNNNNYGYAYGK 3′, is fairly degenerate but will guide future studies to identify other targets of MtrR within the gonococcal genome to clarify further the role of MtrR as a global regulator. To date, MtrR has also been shown to bind the promoters of the *gdhR* and *glnA* genes (21,22). Notably, inspection of their promoter sequences does not reveal a binding site that matches well with the ‘consensus’ sequence, which we report here. Thus, MtrR might employ alternative mechanisms by which it recognizes some of its target sites that require an altered, supercoiling-induced DNA conformation or have a component of indirect readout as has been observed for several transcription factors of the winged Helix-Turn-Helix family that regulate the expression of multiple genes (66). Regardless, there are a number of 5′-YpG-3′ steps within both the *gdhR* and *glnA* operators that likely serve as important nucleation sites for sequence recognition of these operators by MtrR.

**Figure 7.**
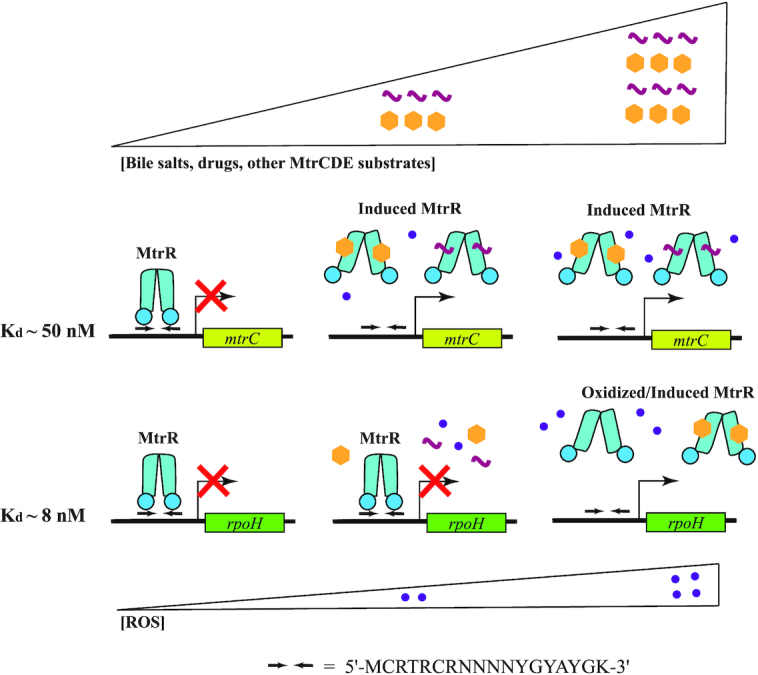
Hypothesized hierarchy of induction of MtrR target genes. The triangles show concentration gradients for MtrCDE substrates including bile salts and drugs as well as for reactive oxygen species (ROS). The schematic represents the regulation of MtrR target genes in response to these concurrent concentration gradients. The shaded rectangles indicate MtrR-regulated genes, *mtrC* and *rpoH*. The corresponding dissociation constants (*K*_d_) are shown to the left of the two operators. The bent arrows represent the transcription start sites while the inverted arrows correspond to the MtrR binding site. The red ‘X’ is present when MtrR is bound to its DNA-binding site to repress transcription. A cartoon of MtrR is shown in blue in its DNA-bound (closed) and induced (splayed) forms. Induction of MtrR by MtrCDE substrates and potential modification of MtrR by ROS are shown in correspondence with their respective concentration gradients. The structure-derived consensus operator sequence is also shown.

Identifying the biochemical recognition mechanisms for two cognate operators of MtrR has significantly broadened our understanding of the biological functionality of MtrR and its clinical implications. Our DNA-binding assays reveal that MtrR has ∼6-fold higher affinity for the *rpoH* operator compared to the *mtrCDE* operator: this suggests that low concentrations of some cytotoxins, i.e., administered drugs or innate response molecules such as certain bile salts, are sufficient for derepression of the multidrug efflux transporter, while the oxidative stress response system controlled by *rpoH* remains more tightly down-regulated by MtrR. Upon reaching a certain, higher threshold concentration of cytotoxin or evoking the production of reactive oxygen species, MtrR will only then be induced from the *rpoH* operator thereby eliciting a full response to these stresses (Figure 7). Furthermore, while bile salts are redox-inert molecules, there is evidence to suggest that the accumulation of these molecules cause *in vivo* disulfide stress in bacteria; however, the mechanism by which this occurs remains unclear (67). Regardless, it is plausible that the accumulation of bile salts over time could result in sequential induction of *mtrCDE* and then *rpoH* as the bacterial cytosol becomes a more oxidizing environment. Additionally, clinical mutations of MtrR that do not abolish DNA-binding but only reduce the affinity of MtrR for its target DNA, such as A39T, may be sufficient for derepression of the *mtrCDE* efflux transporter genes whilst transcription of *rpoH* remains repressed. It is important to note that other accessory factors besides MtrR affect the expression of MtrR-target genes *in vivo*. For example, *mtrCDE* is also regulated by the transcription activator, MtrA, which binds its target operator upstream of the MtrR-binding site in response to cytotoxic molecules to sterically block MtrR-mediated transcription repression of the *mtrCDE* efflux transporter genes (68). Thus, the difference in measured binding affinity between MtrR and the *mtrCDE* and *rpoH* operators may not readily translate under all *in vivo* conditions. This may also explain why our qRT-PCR data revealed comparable increases in expression levels of *mtrC* and *rpoH* when MtrR was deleted or mutated in gonococcal strains.

Importantly, this work also provides a molecular explanation for mechanisms of drug resistance amongst clinical isolates of *N. gonorrhoeae* that harbor often-observed mutations in *mtrR*. Previously, it was unclear how some of these common clinical mutations, which mapped outside of the DNA-recognition HTH motif, conferred drug resistance, in particular those located in the C-terminal multidrug binding domain. The DNA-bound structures of MtrR reveal that steric clash between the allosteric mutation H105Y and residue D68 would disfavour the DNA-bound conformation leading to weaker DNA binding and up-regulation of the MtrR regulon. However, the clinical significance of the H105Y mutation is unclear as strains encoding this mutant MtrR frequently have a co-resident *mtrR* promoter single base pair deletion that abrogates *mtrR* expression (11) (Supplementary Table S5).

Finally, our work further clarifies the role of clinical mutations localized to the DNA-binding domain of MtrR, such as G45D and A39T, and describes the biochemical mechanism by which they confer drug resistance. The mechanism by which D79N might influence levels of resistance is less clear but likely this change to a neutral residue interferes with the movement of α4 and the transition from the induced to the DNA-bound conformation, favouring the former. Clearly, these DNA-bound structures of MtrR will provide molecular scaffolds to explain novel multidrug resistance mutations amongst gonococcal isolates as well as aid investigations to predict the evolution of this protein and emerging mutations in drug resistant strains of *N. gonorrhoeae*.

## DATA AVAILABILITY

The coordinates and structure factor amplitudes for our structures have been deposited in the Protein Data Bank (accession codes: 7JNP and 7JU3).

## Supplementary Material

gkab213_Supplemental_Files
