# Supplementary material for: Structures of Neisseria gonorrhoeae MtrR-operator complexes reveal molecular mechanisms of DNA recognition and antibiotic resistance-conferring clinical mutations
Source: Nucleic Acids Res. 2021 Mar 30;49(7):4155–70. doi: 10.1093/nar/gkab213 (PMC8053128; doi:10.1093/nar/gkab213)
Supplement: gkab213_Supplemental_Files [file gkab213_supplemental_files.zip › Supplementary Material NAR_rgb_FINAL.pdf]

SUPPLEMENTARY MATERIAL

Structures of *Neisseria gonorrhoeae* MtrR-operator complexes reveal molecular mechanisms of DNA recognition and antibiotic resistance-conferring clinical mutations

Grace A. Beggs[1], Julio C. Ayala[2], Logan G. Kavanaugh[2], Timothy D. Read[3,4], Grace M. Hooks[1], Maria A. Schumacher[1], William M. Shafer[2,4,5], Richard G. Brennan[1*]

Department of Biochemistry[1], Duke University School of Medicine, Durham, NC 27710

Departments of Microbiology and Immunology[2], and Medicine[3], and the Emory Antibiotic Resistance Center[4], Emory University School of Medicine, Atlanta, GA 30322

Laboratories of Bacterial Pathogenesis[5], VA Medical Center, Decatur, GA 30033

*Correspondence: richard.brennan@duke.edu

rpoH: 5’ TAC ATA CGT GGT TGT ATG TAA 3’
3’ ATG TAT GCA CCA ACA TAC ATT 5’

mtrCDE: 5’ TAT CCG TGC AAT CGT GTA TGT 3’
3’ ATA GGC ACG TTA GCA CAT ACA 5’

Consensus sequence: 5’ MCR TRC RNN NNY GYA YGK 3’

Figure S1 | DNA-binding sites of MtrR. The *rpoH* and *mtrCDE* target sites. The bases specifically recognized by MtrR are highlighted in green and yellow within the *rpoH* and *mtrCDE* sites, respectively. The consensus sequence recognized by MtrR is also listed whereby R signifies a purine, Y, a pyrimidine; M, A or C; K, G or T; and N any nucleotide.

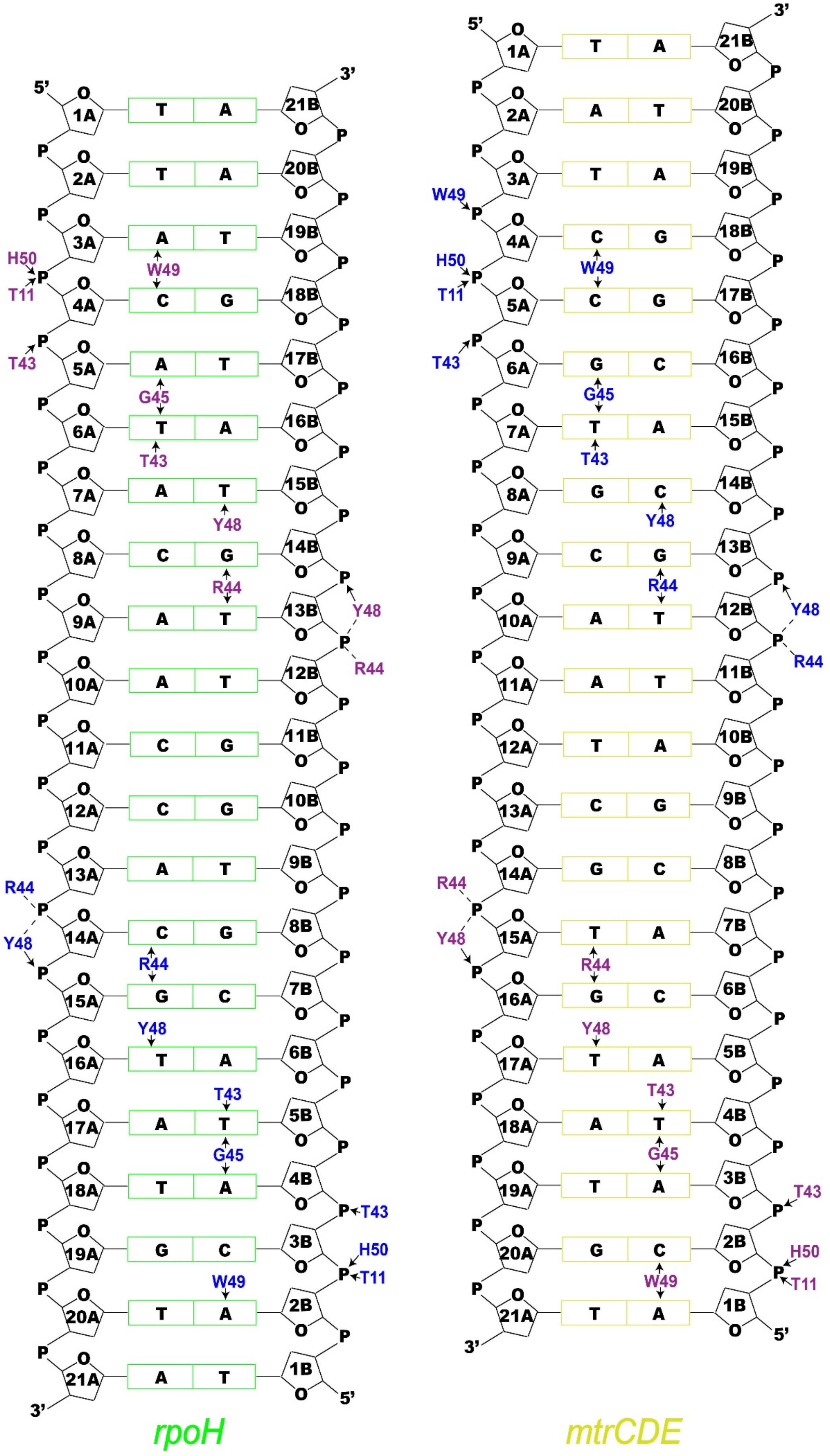

Figure S2 | Overview of MtrR-DNA interactions. Schematic representation of MtrR-DNA contacts. Amino acid residues are displayed in blue or purple correspond to a single MtrR protomer. Interactions between amino acid residues and the DNA operators are indicated by arrows. Water-mediated contacts between amino acid residues and the phosphate backbone of the DNA are represented by dashed lines. The DNA bases for the *rpoH* and *mtrCDE* operators are labelled in green and yellow, respectively.

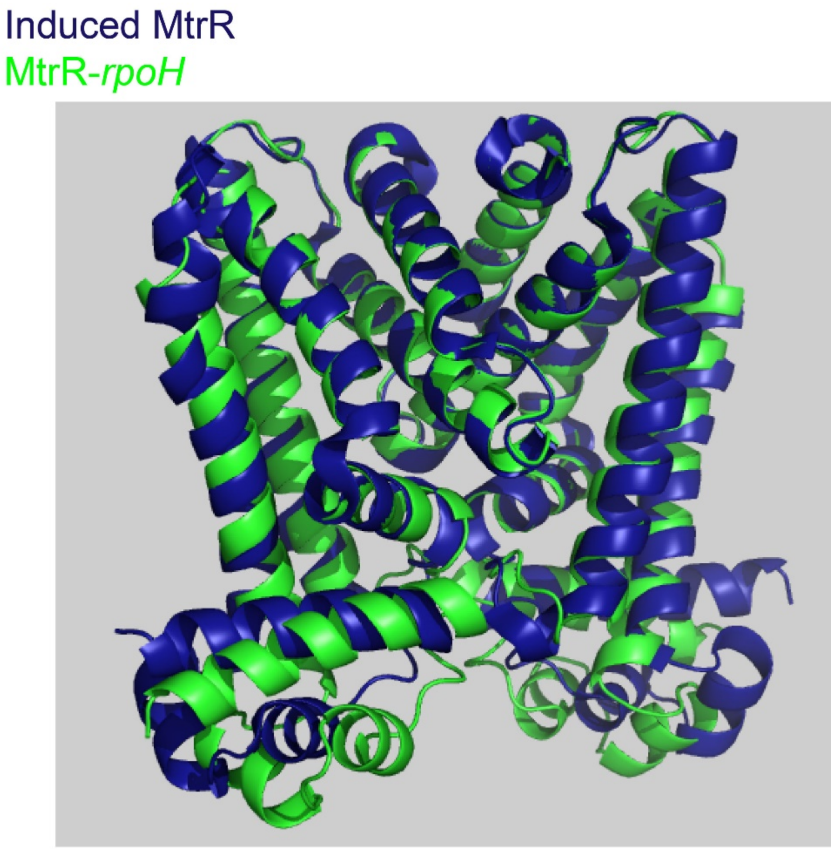

Figure S3 | Alignment of the induced and DNA-bound forms of MtrR. Overlay of MtrR from the MtrR-*rpoH* operator complex structure, which is shown in green, and the induced conformation of MtrR (PDB ID: 6OF0), which is shown in blue.

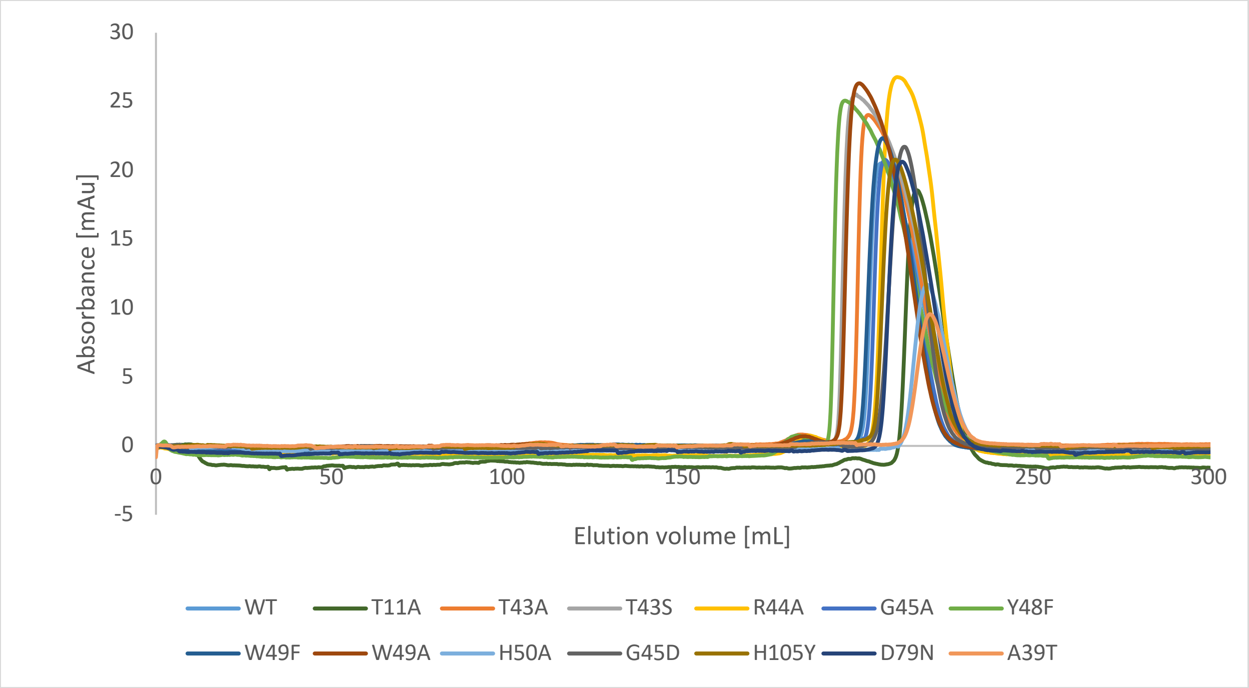

Figure S4 | The size exclusion chromatograms of wild type and MtrR point mutants. The UV absorbance of wild-type MtrR as well as point mutants of MtrR plotted against the elution volume from an S200 size exclusion column. The peak value for all forms of MtrR correspond to the molecular weight of a dimer (~48 kD) and fall within a 25 mL range of each other. Minor variations in the peak values are within the error associated with the size exclusion column.

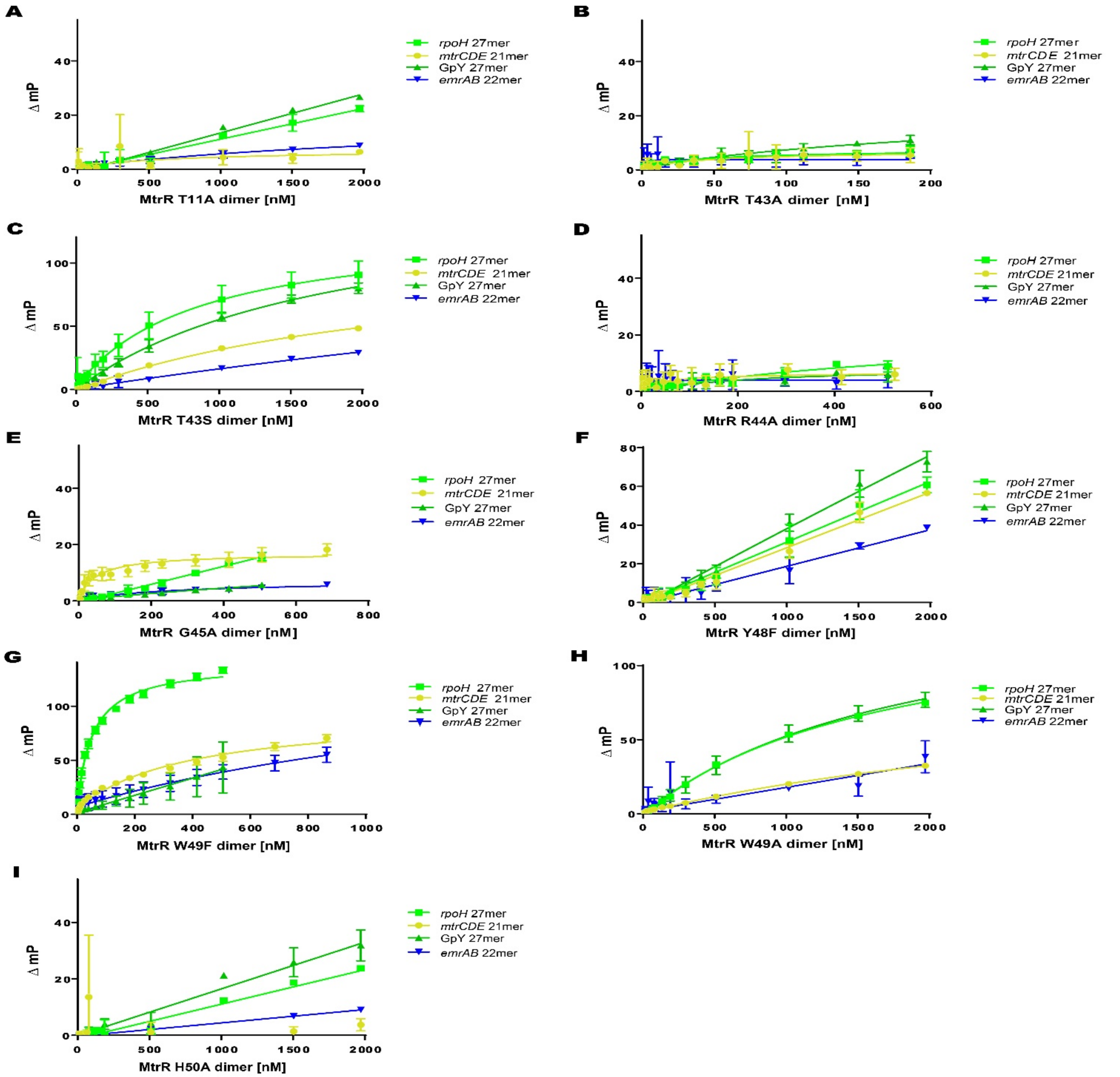

Figure S5 | DNA-binding activity of DNA-binding domain point mutants of MtrR. Fluorescence polarization-based DNA binding assay data with DNA-binding domain point mutants of MtrR and fluorescein-labeled oligoduplexes. The hyperbolic binding isotherms plotting the change in millipolarization versus the initial MtrR mutant dimer concentration are shown for the target oligoduplexes: *rpoH* 27mer (lime green), *mtrCDE* 21mer (yellow), GpY 27mer (kelly green), and *emrAB* 22mer (blue). Data corresponding to the DNA-binding activity of the mutants MtrR(T11A) (**A**), MtrR(T43A) (**B**), MtrR(T43S) (**C**), MtrR(R44A) (**D**), MtrR(G45A) (**E**), MtrR(Y48F) (**F**), MtrR(W49F) (**G**), MtrR(W49A) (**H**), and MtrR(H50A) (**I**) are included.

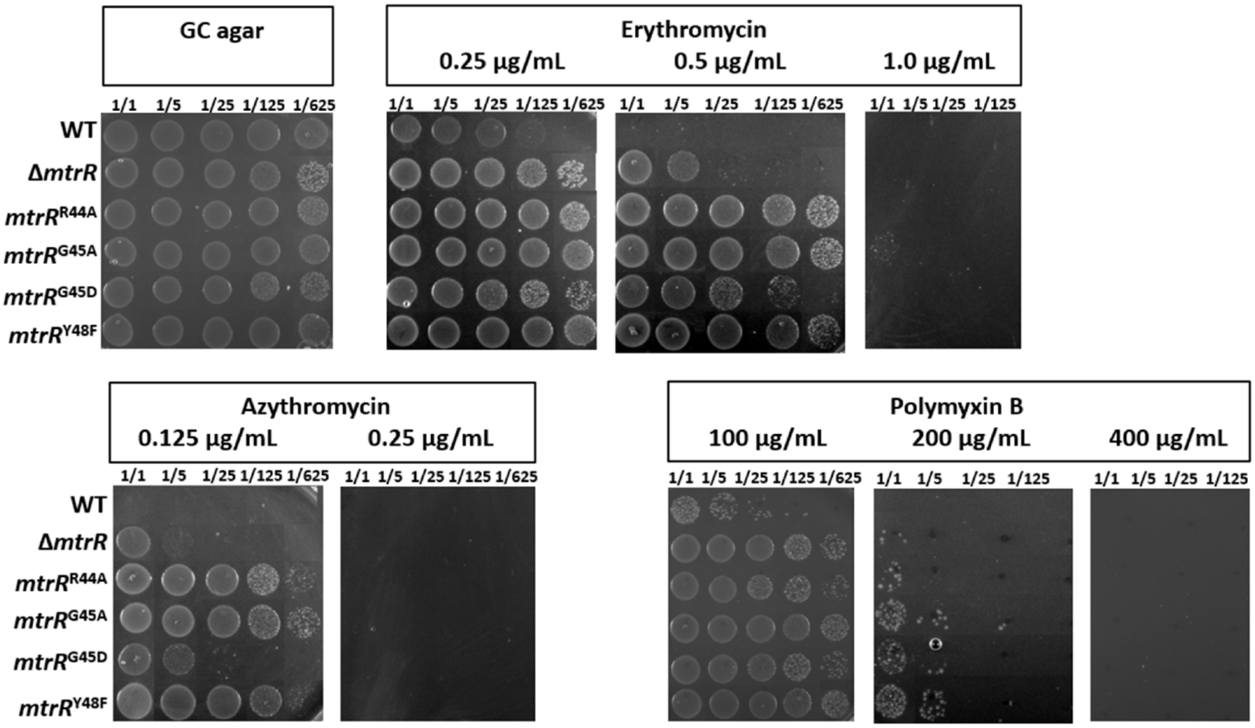

Figure S6 | Effect of *mtrR* missense mutations on antimicrobial susceptibility. Cultures (5 x 10^5 CFU) and serial dilutions of wild type FA19 and mutant strains JF1 (Δ*mtrR*), JC55 (*mtrR* R44A), LK01 (*mtrR* G45A), KH16 (*mtrR* G45D) and LK02 (*mtrR* Y48F) were spotted on GC agar plates containing different concentrations of two macrolides and Polymyxin B. Antimicrobial susceptibility was assessed at 24 h.

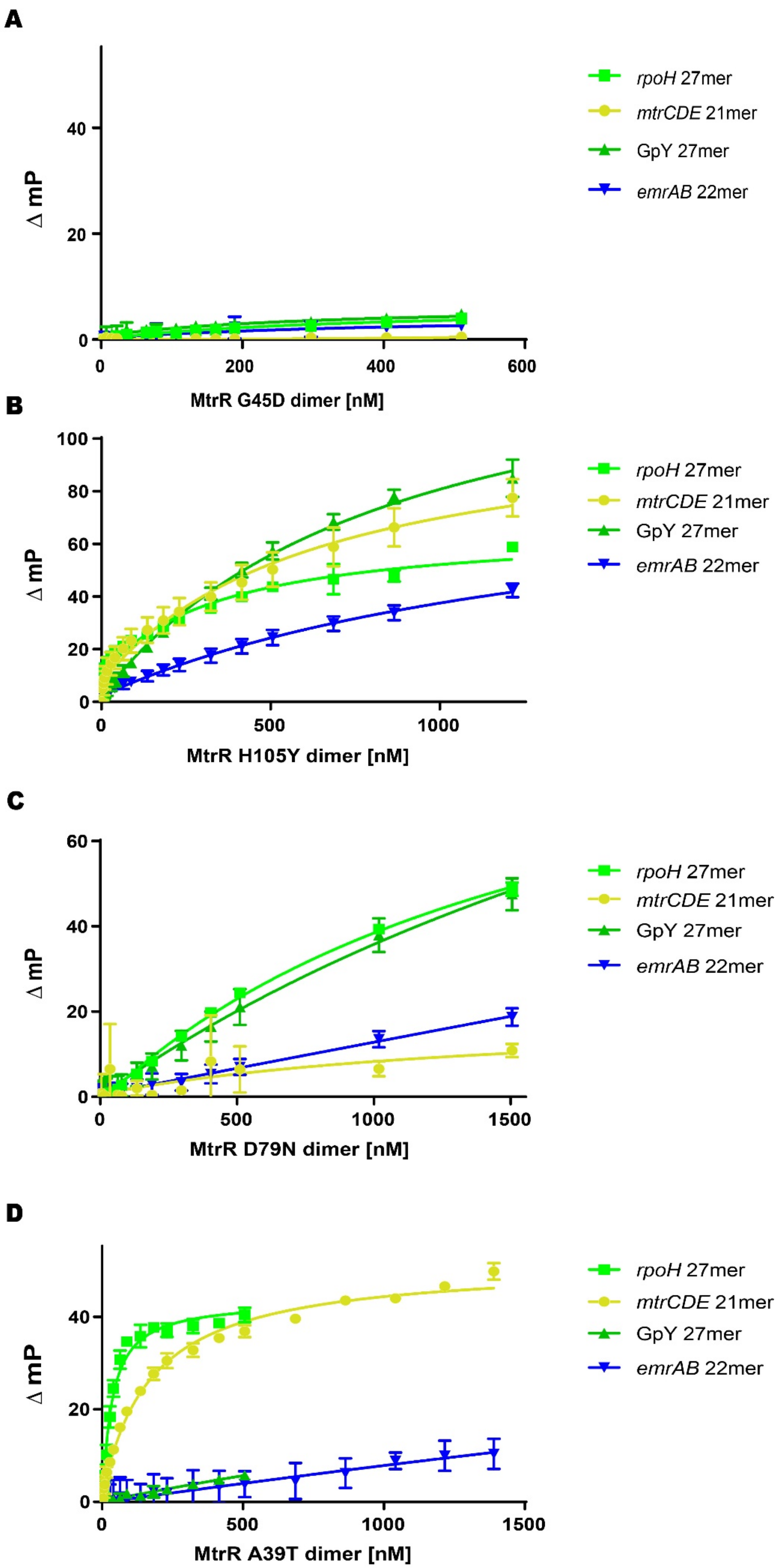

Figure S7 | DNA-binding activity of clinically relevant mutants of MtrR. Fluorescence polarization-based DNA binding assay data with clinically relevant MtrR mutants and fluorescein-labelled oligoduplexes. The hyperbolic binding isotherms plotting the change in millipolarization versus the initial MtrR mutant dimer concentration are shown for each target oligoduplex: *rpoH* 27mer (lime green), *mtrCDE* 21mer (yellow), GpY 27mer (kelly green), and *emrAB* 22mer (blue). Data corresponding to the DNA-binding activity of the mutants MtrR(G45D) (**A**), MtrR(H105Y) (**B**), MtrR(D79N) (**C**), and MtrR(A39T) (**D**) are included.

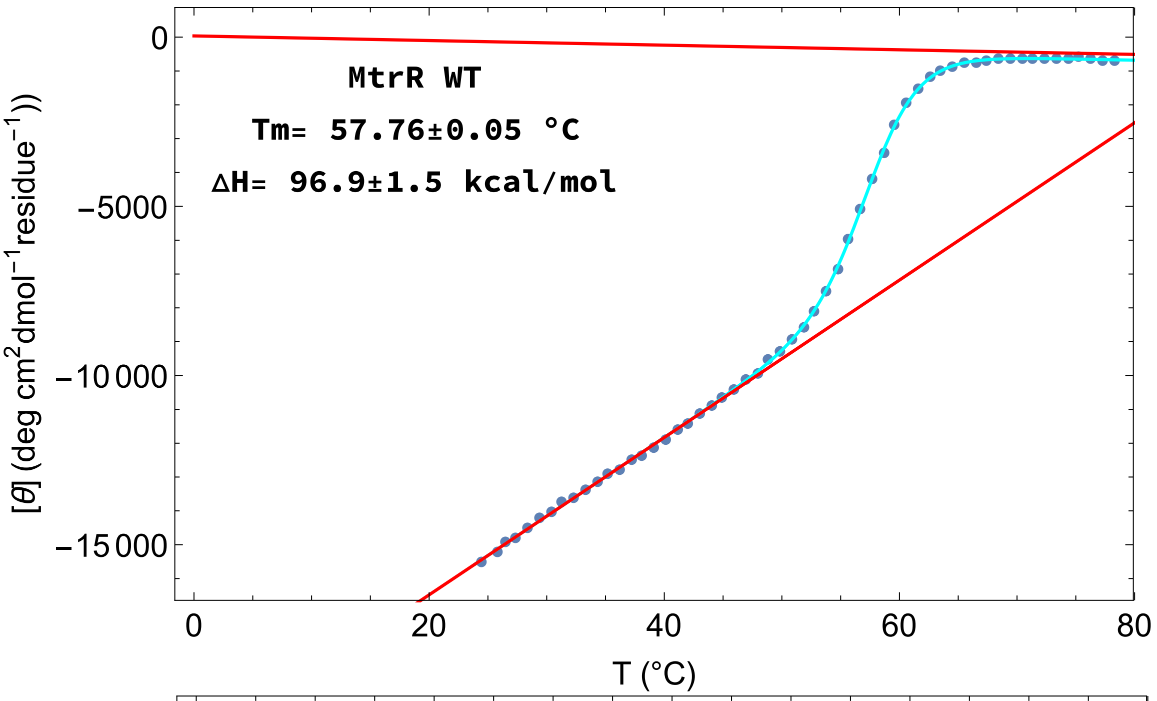

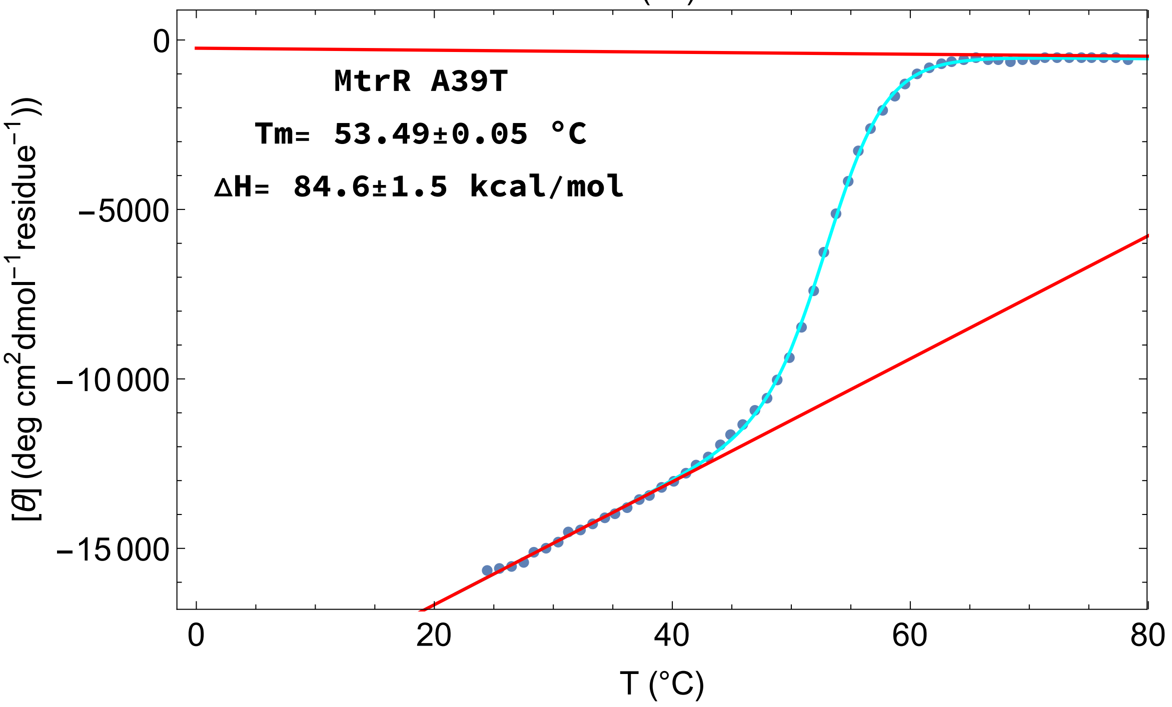

Figure S8 | CD Spectra of MtrR WT and MtrR(A39T). The CD spectra as a function of temperature for MtrR WT and MtrR(A39T) showing the corresponding T_m values.

Table S1: Gonococcal strains

Strains	Description	Reference
FA19 Str^R	FA19 with point mutation in *rpsL*. Streptomycin resistant	[1]
JC55	FA19 Str^R with missense mutation R44A in *mtrR*	This study
LK01	FA19 Str^R with missense mutation G45A in *mtrR*	This study
LK02	FA19 Str^R with missense mutation Y48F in *mtrR*	This study
KH16	FA19 with missense mutation G45D in *mtrR*	[2]
JF1	FA19 ΔmtrR	[3]

References in Table S1

1. Jerse, A.E., et al. *A gonococcal efflux pump system enhances bacterial survival in a female mouse model of genital tract infection.* Infect Immun, 2003. **71**(10): p. 5576-82.
2. Hagman, K.E., et al. Resistance of *Neisseria gonorrhoeae* to antimicrobial hydrophobic agents is modulated by the *mtrRCDE* efflux system. Microbiology, 1995. 141:611-622
3. Folster, J.P. and Shafer, W.M. Regulation of *mtrF* Expression in *Neisseria gonorrhoeae* and Its Role in High-Level Antimicrobial Resistance. *J. Bacteriol*. 2005. 187(11):3713–3720.

Table S2: Plasmids and oligonucleotides for gonococcal genetic transformations and qRT-PCR

Plasmid	Description	Reference
pGAB027RGB	*mtrR* R44A mutant allele cloned in the SspI site of vector pmCSG7	This study
pGAB028RGB	*mtrR* G45A mutant allele cloned in the SspI site of vector pmCSG7	This study
pGAB029RGB	*mtrR* Y48F mutant allele cloned in the SspI site of vector pmCSG7	This study
pGAB024RGB	*mtrR* A39T mutant allele cloned in the SspI site of vector pmCSG7	This study

Oligonucleotide	Sequence
rmpM_qRT_F	AAGCCAAGGTCGCGTAGAAT
rmpM_qRT_R	GGCGCGCAATGAATCCTTAT
mtrC_qRT_F	CGGATTTGGCGCGTTACAAA
mtrC_qRT_R	TAATGCGCGAACGGTTCAGA
mtrR_qRT_F	CTTGTTTGACGCGTTGTTCCA
mtrR_qRT_R	GTGGATGTCGTTGCTTTGCA
recAqFw	AACCTCGAAGTCATTTCCACCGG
recAqRv	TCTGGCATTGGGCGACGGCTTC
KH9#3	GACGACAGTGCCAATGCAACG
CEL1	GACAATGTCATGCGATGATAGG
rpoH_qRT_F	AACGGCAGCCTCGAACAATA
rpoH_qRT_R	GGTGGGACAGGATGAGTTGTT

Table S3: MtrR-DNA contacts shown in Figure 2 and S2.

Figure	Contact: Residue(atom) – base(atom)	Distance (Å)	Interaction
2A	T43(OG1) – G6A(OP2)	3.0	H-bond
2A	R44(NH1) – G13B(O6)	2.5	H-bond
2A	R44(NH1) – T12B(O4)	3.9	H-bond
2A	R44(NH2) – G13B(N7)	2.6	H-bond
2A	R44(NH2) – T12B(C6)	3.7	van der Waals
2A	R44(NH2) – H_2O	2.7	H-bond
2A	Y48(OH) – G13B(OP2)	2.2	H-bond
2A	Y48(OH) – H_2O	2.8	H-bond
2A	T12B(OP2) – H_2O	3.9	H-bond
2A	R44(NH2) – T12B(C6)	3.7	van der Waals
2B	T43(CG2) – T5B(C7)	4.7	van der Waals
2B	R44(NH1) – G15A(O6)	2.6	H-bond
2B	R44(NH1) – C14A(N4)	3.8	H-bond
2B	R44(NH2) – G15A(N7)	3.1	H-bond
2B	R44(NH2) – C14A(C5)	3.4	van der Waals
2B	G45(CA) – T5B(C7)	4.2	van der Waals
2C	Y48(CD2) – C14B(C5)	4.8	π – π interactions
2C	W49(NE1) – C4A(OP2)	3.1	H-bond
2C	W49(CE2) – C4A(C5)	4.0	π – π interactions
2C	W49(CE3) – C5A(C5)	4.2	π – π interactions
2C	H50(NE2) – C5A(OP2)	2.7	H-bond
2C	T11(OG1) – C5A(OP1)	2.3	H-bond

Table S4: Antimicrobial susceptibility of gonococcal strains

	MIC (µg/mL)		
	Erythromycin	Azythromycin	Polymixin B
FA19	0.5	0.125	200
JF1 (ΔmtrR)	1.0	0.25	400
$mtrR^{R44A}$	1.0	0.25	400
$mtrR^{G45A}$	1.0	0.25	400
$mtrR^{G45D}$	1.0	0.25	400
$mtrR^{Y48F}$	1.0	0.25	400

Table S5: Analysis of MtrR substitutions in *N. gonorrhoeae* genomes isolated from around the globe and sequenced by Ezewudo *et al*[a].

MtrR Substitution[b]	Number of strains with substitution	Number of *mtrR* ΔA strains with substitution	Fisher's test P value[c]
A39T	6/55 (10.9%)	0 /23	0.02266*
G45D	20/55 (36.4%)	5/23 (5.7%)	0.009532*
D79N	4/55 (7.3%)	0/23	0.1105
H105Y	26/55 (47.3%)	18/23 (78.3%)	0.001708*

[a]Ezewudo, M.N., Joseph, S.J., Castillo-Ramirez, S., Dean, D., del Rio, C., Didelot, X., Dillon, J.-A., Selden, R.F., Shafer, W.M., Turingan, R.S. *et al.* (2015) Population structure of *Neisseria gonorrhoeae* based on whole genome data and its relationship with antibiotic resistance. *PeerJ*, **3**, e806.

[b]Other positions with known substitutions affecting resistance in other strains (11T,43T,44R,Y48,W49) were not found to be altered in this set.

[c]Test the null hypothesis that the substitutions are distributed evenly across *mtrR* wild-type and ΔA mutant strains. Asterisk means P < 0.05.

Table S6: Summary of MtrR substitutions in *N. gonorrhoeae* genome strains in the USA identified by Gernert *et al*[a.]

***mtrR* mutation**	**Frequency**	**Accompanying mutations (% of cases)**
R44Q	21/300 (7 %)	A39T 20/21 (95.2%) or H105Y 1/21 (4.8%)
A46T	12/300 (4 %)	A39T 10/12 (83.3%) and R130L 1/12 (8.3%)
Y48D	11/300 (3.7 %)	A39T 11/11 (100%)
G45D	37/300 (12.3 %)	Adenine in *mtrCDE* operator deletion (12/37) or A39T 10/37 or H105T/T86A (2/37)
A39T	117/300 (38.9%)	110 have other mutations, only 7 have no other mutations

[a]Gernert, K.M., Seby, S., Schmerer, M.W., Thomas IV, J.C., Pham, C.D., St. Cyr, S., Schlanger, K., Weinstock, H., Shafer, W.M., Raphael, B.H. *et al.* (2020) Azithromycin susceptibility of *Neisseria gonorrhoeae* in the USA in 2017: a genomic analysis of surveillance data. *The Lancet Microbe*, **1**, e154-164.

Table S7: T_m for MtrR WT and MtrR A39T with standard deviation of the curve.

MtrR WT	57.76 ± 0.05 ºC
MtrR A39T	53.49 ± 0.05 ºC
